# GITR-GITRL System, A Novel Player in Shock and Inflammation

**DOI:** 10.1100/tsw.2007.106

**Published:** 2007-05-01

**Authors:** Ludovic Tibor Krausz, Rodolfo Bianchini, Simona Ronchetti, Katia Fettucciari, Giuseppe Nocentini, Carlo Riccardi

**Affiliations:** Dipartimento di Medicina Clinica e Sperimentale, Sezione di Farmacologia, Tossicologia e Chemioterapia, Università di Perugia, IBiT Foundation, Perugia; Polo Scientifico e Didattico di Terni, Italy

**Keywords:** tumor necrosis factor receptor superfamily, tumor necrosis factor superfamily, inflammation, immune response, in vivo model, fusion proteins, T cell modulation, T cells, regulatory T cells, innate immunity, dendritic cells, endothelial cells, inflammatory mediators, cytokines, splicing variant, TRAF pathway, NF-ĸB, Siva, PRMT1, cysteine repeat

## Abstract

Glucocorticoid-induced TNFR-Related (GITR) protein is a member of the tumor necrosis factor receptor superfamily that modulates acquired and natural immune response. It is expressed in several cells and tissues, including T cells, natural killer cells, and, at lower levels, in cells of innate immunity. GITR is activated by its ligand, GITRL, mainly expressed on antigen presenting and endothelial cells. Recent evidence suggests that the GITR/GITRL system participates in the development of inflammatory responses, including shock, either due to early response of neutrophils and macrophages, or together with autoimmune/allergic pathogenesis. The pro-inflammatory role of the GITR/GITRL system is due to: 1) modulation of the extravasation process, 2) activation of innate immunity cells, 3) activation of effector T cells also favored by partial inhibition of suppressor T cells and modulation of dendritic function. This review summarizes the *in vivo* role of the GITR/GITRL system in inflammation and shock, explaining the mechanisms responsible for their effects, considering the interplay among the different cells of the immune system and transduction pathways activated by GITR and GITRL triggering. The hidden aspects about GITR/GITRL function, crucial for treatment planning of inflammatory diseases and shock by modulation of this system is stressed.

**Abbreviations**: CIA, collagen-induced arthritis; COX, cyclooxigenase; CRD, cysteine rich domain; CTLA-4, cytotoxic T cell associated antigen 4; GITR, glucocorticoid-induced tumor necrosis factor receptor related gene/protein; GITR-Fc, soluble GITR fusion protein formed by the extracellular domain of GITR and Fc fragment (dimeric form produced in eukaryotic cell); GITRL, GITR ligand; ICAM-1, intercellular adhesion molecule-1; iNOS, inducible nitric oxide synthase; hGITR, human GITR; LPS, lipopolysaccharide; MMP, matrix metalloproteinase; mGITR, murine GITR; NK, natural killer; NO, nitric oxide; PBMC, peripheral blood mononuclear cells; PMN polimorphonuclear neutrophil; PRMT1, protein arginine N-methyltransferase 1; RPE, retinal pigment epithelial; RA, rheumatoid arthritis; SAO, splanchnic artery occlusion; sGITR, soluble GITR (monomeric form produced in *E.coli*); TNFRSF, TNFR superfamily; TRAF, TNF associated factor; Treg, T regulatory cells

### INTRODUCTION

The concept of inflammation leads to a widening search for the types of cellular and molecular interactions responsible for linking the initial stimulus to the final abnormal function. It has not been possible yet to integrate all this information into a single model for the development of inflammation, but a useful framework is based on the behavior of the immune system. Receptors and soluble mediators produced by local tissue cells and infiltrating inflammatory cells, regulate the progression of inflammation. The nature of local events demands that the soluble mediators act in a spatial and temporally regulated manner.

The first events in response to an inflammatory stimulus mainly involve endothelial cells and innate immunity cells. Endothelial cells upregulate adhesion molecules promoting extravasation of leukocytes. After extravasation and migration, neutrophils (PMNs), monocytes and other leukocytes are activated and release soluble mediators (such as chemokines, cytokines and matrix metalloproteinases-MMPs) which orchestrate the cascade of cellular processes in the microenvironment including further modification in endothelial cells (such as tight junction disorganization and further upregulation of adhesion molecules), apoptosis and tissue remodeling causing, in some cases, fibrosis.

In several cases, the inflammatory response is activated by the reaction to foreign or self antigens, caused by a specific immune response. The principal scheme for integrating this information is based on the classification of the adaptive immune system, and especially the responses of T helper (Th) cells. In this scheme, $CD4^+$ T cell-dependent responses are classified into T helper type 1 (Th1) or type 2 (Th2). An exaggeration of Th2 over Th1 responses to inflammatory stimuli leads to inflammatory disease. The innate immune system, in particular antigen-presenting cells (APC)(dendritic cells, macrophages and also epithelial and B cells) participate to the development of adaptive response. Recent concepts regarding the role of co-accessory receptors and receptor-ligand cross talk definitely contributes to the fine-tuning and orientation of the immune response at a given moment. On the other hand, there is an entire spectrum of cytokines and mediators (prostaglandins, kinins, nitric oxide (NO), chemokines, soluble adhesion molecules, and acute-phase reactants etc.), which contribute to the complexity of interactions. All these effects may render the inflammatory process acute or chronic depending on the persistence of the various signals.

Originally cloned in 1997, glucocorticoid-induced TNFR related (GITR) protein, also called TNFRSF18, is a receptor belonging to the TNFR superfamily selectively activated by its ligand, GITRL[1-8]. In the past few years, there has been much exploration of the GITR-GITRL system as regards the development and function of the immune system and inflammatory response. Nowadays, GITR is generally accepted as a costimulatory molecule on T lymphocytes[9-11]. However, its function is not confined to T cells. In fact, tissue distribution of GITR and GITRL and functional data suggest implication in several functions such as extravasation, activation of innate immunity, skin defense and bone remodeling. Full comprehension of their function is complicated by the peculiar properties of GITR and GITRL including their coexpression in several cells, the possibility of intracellular signaling deriving also from GITRL, the splicing of GITR and their modulation kinetics. This review is an update of the proven and potential role of the GITR-GITRL system, emphasizing its contribution to the inflammatory process and shock development, and the potential therapeutic use of fusion proteins and antibodies modulating the GITR/GITRL system.

## TISSUE DISTRIBUTION OF GITR AND GITRL

### GITR Is Mainly Expressed in T and Natural Killer Cells mGITR mRNA is mainly expressed in immature and mature T cells as shown in Table 1[1,4,12]. mGITR expression in T cells was originally confirmed by flow-cytometric studies[26], and since then several studies have dealt with GITR expression in naïve or activated T cell sub-populations (Table 1). Although some studies consider GITR expression peculiar to Treg cells ($CD4^+CD25^+$ T cells having a regulatory function)[13,27], mGITR and hGITR expression was found on the surface of both $CD4^+$ and $CD8^+$ resting T cells with $CD4^+$ cells (even the $CD25^-$) having a higher GITR expression than $CD8^+$ cells[6,19,27,28]. Expression is similar in single positive cells from the thymus or peripheral lymphoid organs. On the contrary, thymic $CD4^+CD8^+$ cells do not express mGITR[19]. Resting natural killer (NK) and NKT cells express GITR at the levels observed in $CD4^+CD25^-$ T cells[12,22].

Table 1. Distribution of GITR among cells of the immune system in mouse and human

| Cell subset | Activation status | Expression of GITR | References |
|---|---|---|---|
| $CD4^+CD25^-$ T cell (responder function) | Naïve | +++ | [6, 13-17] |
| | Activated | ++++++ | [13-15, 17] |
| $CD4^+CD25^+$ T cell with suppressor function, thymus-derived (Treg) | Naïve | +++++ | [6, 13-15, 17] |
| | Activated | +++++++ | [13-15, 17] |
| $CD4^+CD25^-$ T cell with suppressor function | Naïve | ++++ | [18] |
| $CD8^+$ T cell | Naïve | ++ | [6, 15, 16, 19] |
| | Activated | +++++ | [15, 19] |
| $CD8^+CD28^-$ T cell with suppressor function (antigen specific) | Primed | ++++ | [20] |
| $CD8^+CD25^+$ T cell with suppressor function (thymus derived) | Naïve | ++++ | [21] |
| $CD4^-CD8^-$ thymocyte | Naïve | + | [19] |
| $CD4^+CD8^+$ thymocyte | Naïve | - | [19] |
| Natural killer | Naïve | +++ | [12] |
| | Activated | +++++ | [12] |
| Natural killer T cell | Naïve | +++ | [22] |
| | Activated | ++++ | [22] |
| B cell | Naïve | + | [19] |
| | Activated | ++ | [19] |
| Macrophage / Monocyte | Naïve | + | [12, 19, 23, 24] |
| | Activated | ++ | [19, 23, 24] |
| Plasmacytoid dendritic cell | Naïve | - | [12] |
| | Activated | +++ | [12] |
| Mature dendritic cell | Naïve | - | [12] |
| | Activated | +++ | [12] |
| Mast cell | Naïve | +++ | [25] |
| | Activated | +++ | [25] |
| Polymorphonuclear leukocyte (PMN) | Naïve | +++ | [12, 24] |
| | Activated | +++++ | [24] |
| Eosinophil | | ++ | [12] |
| Basophil | | ++ | [12] |

As regards GITR expression in hematological cells other than T or NK cells, Shimizu et al. described low mGITR expression in B220+ and F4/80+ cells[19], and Shin et al. found low levels of GITR expression in macrophages and a macrophage-derived cell line[29,30]. Weak expression of GITR on APC cells is further confirmed by other studies[11,12,19]. GITR is expressed on non-activated bone marrow derived mast cells[25]. Expression levels of mouse and human GITR in hematological cells are summarized in Table 1.

Some non-lymphoid tissues, such as lung, kidney and small intestine express mGITR mRNA (Table 2). GITR expression was detected also on osteoclast precursor cells, keratinocytes and retinal pigment epithelial (RPE) cells [31-33]. A similar (though not perfectly matched) pattern of expression was described in humans. hGITR mRNA was expressed at a good level in lung and, at a low level, in brain, kidney and liver[3,4]. It was also found in a colorectal adenocarcinoma cell line[3]. In summary, GITR is mainly expressed in hematological cells, but there is some evidence that it is also expressed in non-lymphoid tissues. Other TNFRSF members sharing structural properties with mGITR, such as 4-1BB, although expressed mainly in lymphoid organs are also found in some non-lymphoid cells such as lung[34].

Table 2. Tissue distribution of mGITR evaluated by RNase protection assay (RPA) (Bianchini et al., unpublished data)

| Tissue | Expression of GITR* |
|---|---|
| Lung | 13 |
| Bone marrow | 10 |
| Kidney | 8 |
| Small intestine | 6 |
| Liver | 3 |
| Ovary | 2 |
| Brain | 4 |
| Testis | 3 |
| Adipose tissue | 2 |
| Muscle | 4 |
| Heart | 3 |
| Thymus | 80 |
| Spleen | 100 |

* GITR expression was normalized using GAPDH as house-keeping gene and referred to spleen expression considered equal to 100.

### GITR Expression is Upregulated in Activated Cells

After T cells are activated, both murine and human T cells strongly upregulate GITR expression at mRNA and protein level[1,3,4,15,35]. After T cell receptor (TCR) triggering, GITR expression is induced at 6 h and peaks within 24 h[15]. mRNA levels remain upregulated for at least 3 days from activation[1]. Interestingly, in an *in vivo* murine model, GITR, 4-1BB and OX40 were upregulated in tumor-specific T cells that promote regression of SP2/0 myeloma tumor[36]. hGITR was also upregulated in $CD4^+$ T helper cell subpopulation of patients with non-infectious uveitis, a Th1 cell mediated autoimmune disease, and correlated positively with active uveitis[35].

Following NK and NKT activation, GITR is strongly upregulated[12,22]. GITR is also present in inflamed blood vessel endothelial cells[23] and lipo-polysaccharide (LPS) activated immature dendritic cells (DC) injected subcutaneously upregulates GITR[37]. In summary, several cells participating to the inflammatory process upregulated GITR expression after activation suggesting that GITR is involved in the modulation of inflammation.

### GITR is Expressed at High Levels in T Regulatory (Treg) Cells and Other Suppessor T cells

Over the last ten years, the concept of specialized suppressor T cells, capable of controlling immune responses and preventing autoimmune diseases (T regulatory cells, Treg cells) has been well established[38]. However, markers capable of distinguishing genuine Treg cells from recently activated responder T cells are few and somewhat uncertain. In a search for novel Treg markers, 2 different studies found that freshly isolated murine $CD4^+CD25^+$ Treg cells have higher mRNA and protein levels of GITR than conventional $CD4^+CD25^-$ T cells (responder cells)[13,19]. At the same time, another study reached the same conclusion after comparing $CD4^+$ T cell clones with suppressor function and Th1 and Th2 clones with responder function[17]. Human Treg cells ($CD4^+CD25^+$) also expressed GITR high levels[35,39], and GITR was overexpressed in a human thymic $CD8^+$ sub-population with suppressor function ($CD8^+CD25^+$)[21]. Treg cell activation increases GITR expression[13,19]. In human $CD4^+CD25^+$ suppressor clones, suppressive activity correlated in full with the intensity of GITR staining and intracellular cytotoxic T cell associated antigen 4 (iCTLA-4), a marker of fully active Treg cells[40]. Some *in vivo* studies have provided further evidence that GITR is overexpressed in T cells with suppressor function. In murine T cells from tolerated skin grafts, expression of Treg markers (including GITR) was higher than in T cells from rejected skin grafts[17,41]. In human decidua, expression of GITR and OX40 is higher in cells positive for iCTLA-4 ($CD4^+CD25^+iCTLA\text{-}4^+$) than in negative ($CD4^+CD25^+iCTLA\text{-}4^-$) and responder T cells ($CD4^+CD25^-$)[42]. Finally, $CD4^+CD25^{bright}$ T cells in the human intestinal lamina propria and in the joints of patients with the remitting form of juvenile idiopathic arthritis present high levels of GITR on their surface[43,44].

Thus, there is overwhelming evidence that GITR is one of the few markers of cells with suppressor activity, and a practical demonstration is provided by studies in which GITR has been used to sort regulatory cells. For example, Shimizu et al. demonstrated that T cells depleted of $GITR^{high}$ T cells cause autoimmune gastritis in nude mice, suggesting that $GITR^{high}$ cells act principally as suppressor cells[19]. In addition, studying an *in vivo* murine model, Uraushihara et al. hypothesized that GITR is a more representative marker of Treg cells than CD25[18], and demonstrated that $CD4^+$ T cells with high levels of GITR on their surface ($GITR^{high}$) exert suppressor activity independent of CD25 expression. They suggested that the $CD25^-GITR^{high}$ cells are suppressor T cells with a memory function, while the $CD25^+GITR^{high}$ cells are Treg cells with an effector function[18].

A turning point in the definition of GITR as a Treg marker is represented by studies correlating GITR to forkhead box protein p3 (Foxp3), a transcription factor determinant for acquisition and maintenance of the Treg phenotype[45]. In fact, Foxp3 seems to be a negative regulator of IL-2 and IFNγ as a transcriptional repressor by histone deacetylation[46]. Foxp3 also binds the promoter regions of GITR, CD25 and CTLA-4 but acts as a histone acetylator here and therefore a coactivator of the mentioned genes[46]. Among these genes, GITR seems to be more sensitive to Foxp3 regulation. In fact, in Foxp3 transgenic mice, $CD4^{+}CD25^{-}$ cells show suppressive activity and express high levels of GITR[47]. Furthermore, Foxp3 transfection of naive $CD4^{+}$ cells causes GITR upregulation[48]. Downregulation of Foxp3 expression in human type 1 regulatory T cells (Tr1) causes the loss of suppressor activity together with the loss of GITR and iCTLA-4 expression but not CD25 expression[49]. In line with these results, virtually all $Foxp3^{+}CD4^{+}$ cells are $GITR^{+}$ even if not all $GITR^{+}CD4^{+}$ cells are $Foxp3^{+}$[50]. In addition, when cocultured with activated endothelial cells, $CD4^{+}$ effector T cells can generate suppressor T cells which are $CD25^{+}iCTLA\text{-}4^{+}GITR^{high}$ [51], suggesting that $GITR^{high}$ cells deriving from effector T cells may have acquired the suppressive phenotype, at least in some conditions. A similar conclusion was reached by a study using thrombospondin, a natural anti-inflammatory extracellular matrix protein, showing that this protein generates peripheral Treg cells in humans, expressing GITR, CTLA-4, OX40, independent of TGFβ, from resident $CD25^{-}$ naive or memory cells[52].

Taken together these studies indicate that: 1) GITR is a marker of cells with suppressor function; 2) GITR seems to be a more reliable marker than CD25 because is present in regulatory cells that are $CD25^{-}$; 3) GITR is operationally more useful than iCTLA-4 and Foxp3 since there is no need to permeabilize and kill cells for staining.

While the use of GITR as a Treg cell marker seems reasonable and even advisable when studying cells from healthy animals (in which the immune system is not reacting against antigens), in human diseases (particularly chronic) it may be misused. In fact, GITR is upregulated in effector T cells during activation, reaching expression levels comparable to Treg cells. These observations (common to other Treg markers) might hamper the use of GITR as a Treg cell marker, particularly in chronic diseases. For example, in $CD4^{+}$ cells from $Foxp3^{-/-}$ mice lacking suppressor cells, GITR expression is much higher than that observed in $CD4^{+}$ cells from wild type mice[45]. In this case, GITR seems to be a better marker for activated T cells than for Treg cells. Therefore when sorting cells with suppressive activity, we propose at least a two-marker system including Foxp3 and GITR[18,53].

The above-cited studies suggest that GITR is expressed in several kinds of cells with suppressor activity. However, Every et al. demonstrated that $CD4^{+}$ T cells preventing experimental autoimmune diabetes are not defined by Foxp3 and GITR markers[54], suggesting that not all regulatory cells are $GITR^{+}$ or that GITR is expressed only in suppressor T cells characterized by Foxp3 expression.

### Regulation of GITR Expression by Glucocorticoids: A Controversial Matter

When originally cloned, GITR was found upregulated in a hybridoma T cell line treated with glucocorticoids[1]. GITR is, however, only slightly upregulated in T cells in primary cultures treated with glucocorticoids[55,56], and in Treg cells after dexamethasone treatment[55]. GITR expression in T cells is not decreased in glucocorticoid receptor knock out mice[56] and, in humans, is not upregulated in T cells after glucocorticoid treatment[13,19]. Therefore, the relationship between GITR and glucocorticoids remains controversial and seems to have slight functional meaning, if any.

### Tissue Distribution of GITRL

When considering studies evaluating GITRL expression at protein level, it results that GITRL is expressed in professional and non-professional APCs, including unstimulated myeloid DC subsets, plasmacytoid DC precursors (pDC), B cells and monocytes[2,3,5,7,12,28]. Following a preliminary observation suggesting that GITRL is expressed in human endothelial cells[4], a recent array study demonstrated that GITRL is one of the 20 genes more differently expressed in endothelial cells compared to a panel of cells from other tissues. In particular, GITRL is expressed at good levels in microvascular-derived primary cultures, levels higher than in unstimulated APCs[57]. GITRL is also expressed in mouse endothelial cells as observed by Cuzzocrea et al. (personal communication). Not surprisingly, an analysis of EST (expressed sequence tag) expression suggests that GITRL is mainly found in the connective tissue (Mm268623 NCBI, Unigene, EST profile viewers).

Cells different from APC and endothelial cells express low levels of GITRL. According to an expression panel obtained with microarray technology, GITRL is expressed at low levels in T cells, PMNs and NK cells[12], but it must still be ascertained if the low mRNA level observed determines sufficient protein expression to have a functional meaning. GITRL is also expressed in osteoclast precursors, skin, keratinocytes and retinal pigment epithelium, which is an immunologically restricted area, where GITRL seems to modulate immune privilege vs. inflammation[31-33,58]. EST expression suggests that GITRL is expressed in some parts of the central nervous system (T1DBASE TNFSF18 Tissue Expression).

Interestingly, GITRL expression is strongly increased during inflammation, mainly in APCs and endothelial cells. In response to proinflammatory stimuli, GITRL is rapidly upregulated (peak within 2-24 hours) and declines in 1-2 days to the initial or even lower levels[7,11,16,57]. pDCs, stimulated with viruses, overexpress GITRL[12] and human monocytes stimulated with staphylococcal enterotoxin B (SEB) become GITRL$^+$[59]. This is confirmed by *in vivo* experiment showing that 24-48 h subsequent to ocular herpes simplex virus 1 (HSV-1) infection, GITRL expression is increased in APCs of draining lymph nodes[60]. Not all pro-inflammatory stimuli promote GITRL ligand upregulation. For example, in endothelial cells, GITRL is upregulated by IFNα and IFNβ, and not by proinflammatory cytokines and LPS. In addition, T cells upregulate GITRL after activation[16] or DEX treatment (Figure 1).

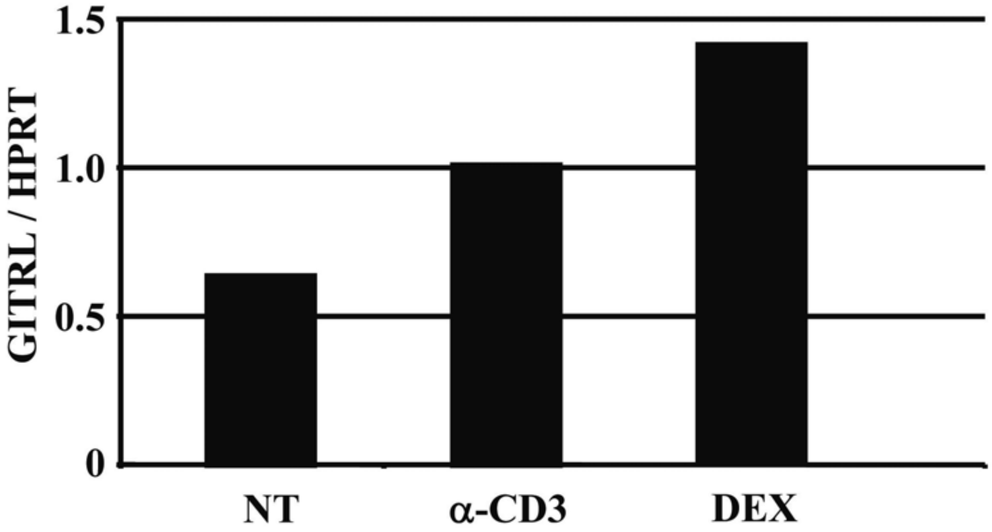

**FIGURE 1.** GITRL expression in T cells after triggering of TCR or treatment with glucocorticoids

GITRL is upregulated on CD4$^+$ T cells by TCR triggering (α-CD3 antibody) or Dexamethasone treatment (DEX) as demonstrated by quantitative RT-PCR experiments. HPRT house keeping gene was used as control (unpublished data).

In conclusion, the widespread distribution of GITRL, its expression in endothelial cells and the upregulation upon specific stimuli suggest that GITRL is involved in the development of inflammatory process.

### THE ROLE OF THE GITR-GITRL SYSTEM IN *IN VIVO* MODELS OF ACUTE INFLAMMATION WITH RAPID ONSET, INCLUDING SHOCK

GITR gene deficient ($GITR^{-/-}$) mice were a useful tool for studying the role of GITR/GITRL system in inflammation. The first study demonstrating a link between the GITR/GITRL system and acute inflammation was conducted on a mesenteric infarction model performed clamping the celiac and superior mesenteric arteries for 45 minutes and called splanchnic artery occlusion (SAO) model[61]. In this model, the survival rate of $GITR^{-/-}$ mice was dramatically higher than that of $GITR^{+/+}$ mice (70% vs. 5%). Decreased mortality of $GITR^{-/-}$ mice correlated with a much lower infiltration of inflammatory cells in the mucosa (with particular reference to PMN), reduction of apoptosis at villus tips and reduction of lipid peroxidation, a marker of oxidant molecules and free radical production. Moreover, in $GITR^{-/-}$ mice there was a lower production of cytokines such as tumor necrosis factor alpha (TNFα), as early as 1 hour following SAO procedure. At the same time, the adhesion molecules P-selectin, E-selectin and intercellular adhesion molecule-1 (ICAM-1) were upregulated in endothelial cells of $GITR^{+/+}$ mice but upregulation was much less efficient in $GITR^{-/-}$ mice, suggesting that the GITR/GITRL system favors PMN infiltration and leukocyte rolling, modulating adhesion molecules during the inflammatory process. Of note, ICAM-1 is expressed at basal levels in both $GITR^{+/+}$ and $GITR^{-/-}$ mice, suggesting that the GITR/GITRL system does not interfere with basal expression of adhesion molecules.

Involvement of GITR in acute inflammation was confirmed by the lower inflammatory response of $GITR^{-/-}$ mice to carrageenan administration in the pleurisy model (carrageenan-induced lung inflammation)[23]. In this model, mice develop an inflammatory response promoting pleural exudation and lung inflammation, 2-8 hours following carrageenan injection in the pleural cavity. In $GITR^{-/-}$ mice, pleural exudate, containing less pro-inflammatory cytokines and a lower number of proinflammatory cells, was reduced of about 50%. The decreased number of cells in the pleural cavity concerned all subsets of pro-inflammatory cells and correlated with decreased lung injury (including apoptotic cells) and inflammatory cell infiltration (with particular reference to PMN) in lungs of $GITR^{-/-}$ mice. Moreover, lower expression of inducible nitric oxide (NO) synthase (iNOS) and cyclooxigenase-2 (COX-2) was found in the lungs of $GITR^{-/-}$ mice, together with lower levels of NO-derivative products, nitrotyrosine, and prostaglandin $E_2$ ($PGE_2$). Finally, adhesion molecules were less upregulated in $GITR^{-/-}$ mice compared to $GITR^{+/+}$ mice, similar to what was observed in the SAO model. Interestingly, co-administration of carrageenan and a fusion protein, formed by the extracellular domain of mGITR fused to human IgG1 Fc fragment (GITR-Fc), in $GITR^{+/+}$ mice decreased pleural infiltration of macrophages and lung infiltration of PMN to levels comparable to those observed in $GITR^{-/-}$ mice injected with carrageenan alone, suggesting that the differences observed between $GITR^{+/+}$ and $GITR^{-/-}$ mice were mainly due to the lack of GITR triggering by its ligand.

However, other *in vivo* models suggest that the triggering of GITRL (supposed to be capable of reverse signaling) positively modulated the inflammatory response. Intraperitoneal injection of recombinant monomeric GITR produced in *E. coli* (sGITR), caused inflammation of the peritoneal membrane and spleen as suggested by increased myeloperoxidase activity in the peritoneal membrane, PMN and monocyte infiltration, with later development of tissue damage, and enlargement of the spleen red pulp[28]. Infiltrating PMNs produce oxygen derivatives, serine proteases and zinc MMPs that promote tissue injury. Another study demonstrated that intraperitoneal injection of sGITR upregulates MMP-9 production[62]. Even if the above data may seem in contrast with attenuation of pleurisy by GITR-Fc fusion protein, note that the reagents used were different. Moreover, it is possible that abolishing GITR triggering is useful during inflammation, while GITRL triggering at levels higher than those obtained with physiological triggering may have a pro-inflammatory significance in the healthy animal. Further studies using $GITR^{-/-}$ mice and $GITRL^{-/-}$ mice (the latter, however, are still not available) will help to discriminate the effect of GITR and GITRL triggering.

### THE ROLE OF THE GITR-GITRL SYSTEM IN *IN VIVO* MODELS OF SLOW DEVELOPMENT INFLAMMATION

#### Inflammatory Diseases in Which Innate Immunity Plays a Significant Pathogenetic Role

GITR$^{-/-}$ mice were studied during the development of lung injury caused by bleomycin instillation, a pro-inflammatory stimulus leading to pulmonary fibrosis[63]. While bleomycin instillation caused death and weight loss in GITR$^{+/+}$, neither death nor weight loss was observed in GITR$^{-/-}$ mice, suggesting that GITR$^{-/-}$ mice were less sensitive to bleomycin treatment. In fact, in these mice the degree of lung infiltration and edema formation was reduced (about one third), 7 days after bleomycin intratracheal instillation. Histological evidence of lung injury was also less. In lungs of GITR$^{-/-}$ mice myeloperoxidase activity and expression was about five fold less than in GITR$^{+/+}$ mice. As a consequence of fewer inflammatory cells in lungs, cytokine production and nuclear factor kappa B (NF-κB) activation were reduced. Very similar results were obtained in GITR$^{+/+}$ mice co-treated with bleomycin and a very low dose of GITR-Fc, administered by a mini-osmotic pump releasing the fusion protein over the whole 7 day-observation period.

Colon inflammation by 2,4,6-trinitrobenzene sulfonic acid (TNBS) delivered intrarectally is a murine model of inflammatory bowel diseases (IBD), a relatively common inflammatory disease of the gastrointestinal tract supposedly deriving from dysregulation of CD4$^{+}$ T helper cells and the innate immune system. GITR$^{-/-}$ mice were less sensitive to TNBS-induced colitis compared to GITR$^{+/+}$ mice, as suggested by macroscopic (survival, weight, clinical score), and microscopic (histological score) parameters and cytokine production[24]. Administrating GITR-Fc partially protected TNBS-treated GITR$^{+/+}$ mice from colitis similar to what observed in TNBS-treated GITR$^{-/-}$ mice. The role of innate immunity in the development of TNBS-induced colitis was demonstrated using immunodeficient SCID mice that develop colitis in response to intrarectal instillation of TNBS, roughly comparable to that seen in wild-type mice. Administrating GITR-Fc to SCID mice partially prevented inflammation induced by TNBS, suggesting that GITR triggering has a role in the development of TNBS-induced colitis also in immunodeficient mice.

The epidermis is a tissue where the GITR-GITRL system seems to play an anti-inflammatory and protective role. In fact, GITR$^{-/-}$ mice exposed to UVB, demonstrated two times more apoptotic cells compared to GITR$^{+/+}$ mice[31]. This was confirmed in *in vitro* studies on keratinocytes from GITR$^{+/+}$ and GITR$^{-/-}$ mice. Moreover, GITR expression is downregulated in response to UV treatment, but when overexpressed, it protects cells from UVB-induced death. In conclusion, GITR protects keratinocytes from cells death, a feature of inflammatory response. The anti-inflammatory role of GITR in the skin is also emphasized by a study on human skin cells, demonstrating that suppressor T cells expressing high levels of GITR proliferate in the skin and may limit skin inflammation[64].

#### Inflammatory Diseases With an Allergic and Autoimmune Pathogenesis

*In vitro* studies clearly demonstrate that GITR potentiates T-mediated immune response. This is confirmed by studies on *in vivo* models where diseases are due to the adaptative immune response. Since in some of these models the inflammatory response is relevant, they are briefly summarized below.

Rheumatoid arthritis (RA) is an autoimmune disease with a substantial inflammatory reaction during both the acute and chronic phases. In the model of collagen-induced arthritis (CIA), GITR$^{-/-}$ mice had a lower incidence of CIA and less joint injury compared to the control mice[65]. Clinical evidence correlated with a lower level of PMN infiltration and pro-inflammatory products, including chemokines, cytokines, iNOS and COX-2. Reduced susceptibility to CIA was due to GITR modulation of effector and Treg cell function. At the same time, another study demonstrated that anti-GITR Abs exacerbate CIA, as ascertained by clinical scores and cytokine production[66], so confirming the role of the GITR/GITRL system in this murine model. Involvement of GITR in RA disease is further suggested by a study in which paired samples of synovial cells and PBMC from rheumatoid arthritis patients were analyzed for GITR, OX40, Foxp3 and CTLA-4 (extra and intracellular). These proteins resulted in an increase in the synovium compared to the PBMC, suggesting that Treg phenotype cells tend to accumulate in the synovial fluid of RA patients, and that GITR is involved. Of note, the number of $CD25^+$ cells was comparable[67].

Administration of anti-GITR Ab to OVA-sensibilized/challenged mice exacerbated allergic airway inflammation in this asthma model[66]. Bronchoalveolar eosinophilia, peribronchial and perivascular inflammation was increased compared to control mice. Serum anti-OVA IgE and total IgE was enhanced, while IgG1 and IgG2a was unaltered. These results suggest that in this case both Th1 and Th2 type responses are upregulated.

*In vivo* administration of anti-GITR Ab aggravates autoimmune thyroiditis (EAT) induced by thyroglobulin in a Hashimoto model, inhibiting tolerance induction and abrogating established tolerance, resulting in increased mononuclear infiltration of the thyroids and autoantibody production. GITR engagement induces autoreactive T cell development and escape from Treg suppression [68], as discussed below.

Altogether, the data presented suggest that GITR signaling increases the expression of those mediators involved in the inflammatory process.

### Inflammatory Diseases Deriving From Response To Viruses

The relationship between viruses and TNFRSF members is well known and it was hypothesized that the low level of interspecies conservation of their extracellular domain is due to the crucial role of TNFRSF members in the struggle against viruses[69]. In a very recent study comparing TNFRSF members, GITR/GITRL pair was the only strictly species-specific one[8], suggesting that GITR may be one of the TNFRSF members more directly involved in the response against viruses. In fact, some *in vivo* studies have recently demonstrated that GITR triggering potentiates immune response against viruses[60,70,71].

Modulation of the GITR/GITRL system may be helpful also in controlling virus-induced inflammatory reaction. A model of inflammation-derived lesion following virus infection is corneal blindness caused by herpes simplex virus (HSV) infection. Effector $CD4^+$ cells, modulated by Treg cells, orchestrate the immunopathological lesions. In planning the study of GITR activation effect on this model, the authors anticipated that treatment with agonistic anti-GITR Ab would cause more severe keratitis either because of negative modulation of Treg suppressive activity or due to the co-stimulatory effect of GITR that could enhance T cell effector function[60]. However, while anti-GITR treatment did enhance HSV-specific T cell immunity (as shown by increased IL-2 and IFNγ production in lymph nodes and spleen), it also reduced virus-induced angiogenesis and stromal keratitis. This effect was explained by 2 anti-GITR-induced effects: 1) decreased infiltration of $CD4^+$ cells in corneas (about half compared to Ig-treated mice), evaluated 10 and 15 days after infection, 2) a five-fold lower production of MMP-9, a matrix-degrading enzyme involved in virus-induced angiogenesis, evaluated 2 and 13 days after infection. Thus, the GITR/GITRL system participates in modulating the inflammatory response caused by virus infection, but contrary to expectations, it plays an anti-inflammatory role.

### HOW THE GITR-GITRL SYSTEM HAS A ROLE IN THE INFLAMMATORY PROCESS AND SHOCK: FROM THE EVIDENCE TO THE CELLULAR MECHANISMS

#### GITR and GITRL: Multifaceted Players in Several Systems

The role of the GITR/GITRL system in modulating the inflammatory response is evidenced by the above-referred *in vivo* data and seems to be crucial both in the early phase and in sustaining the inflammatory process. This is due to the determinant role of the GITR/GITRL system in 4 different aspects of inflammation: 1) extravasation process, 2) production of inflammatory mediators, 3) production of cytokine, 4) activation of effector T cells. Though the effects of the GITR/GITRL system are impressive, it is not always clear how these effects are potentiated by pharmacological treatment.

The main confusing factor is the possibility that GITRL not only represents the molecule able to triggers GITR, but can activate signals (called reverse signaling) in the cells where it is expressed following GITR binding. Reverse signaling of TNFSF members was speculated when the high interspecies conservation of their short cytoplasmic domains was seen[72]. High interspecies conservation is observed in GITRL also. Among the different studies suggesting the existence of reverse signaling by GITRL, two convincingly support this, even if, in our opinion, a definite demonstration will be accomplished by working with $GITR^{-/-}$ cells. The first study demonstrated that GITRL signaling causes cell cycle arrest and apoptosis in murine macrophages[73], the second that GITRL signaling stimulates osteoclast differentiation[32]. These studies also demonstrate that GITR fusion proteins, but not anti-GITRL Ab can trigger GITRL.

The potential GITRL reverse signaling is a confusing factor because several cells, including macrophages, PMNs, DCs and activated T cells express both GITR and GITRL every time a fusion protein is used it can elicit opposite effects on GITR and GITRL. For example, when an agonistic GITR-Fc is used, 2 effects are possible: 1) inhibition of GITR activation by endogenous GITRL, 2) activation of GITRL. Also in $GITR^{-/-}$ mice cells lack both GITR and GITRL signaling, since GITRL, present in $GITR^{-/-}$ mice, is not activated by GITR. The potential effects of GITR and GITRL triggering in macrophages are summarized in Figure 2.

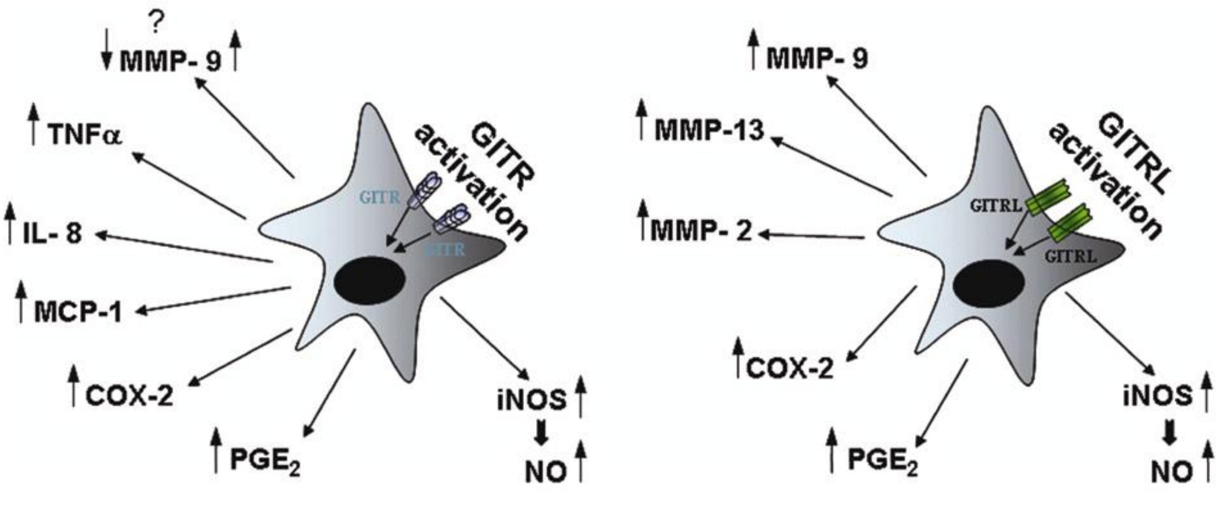

**FIGURE 2.** Potential effects of GITR and GITRL triggering on macrophages.

GITR gene encodes several alternative spliced products, 2 of which (GITRD and GITRD2) are soluble, as presented in detail in a following paragraph. The levels of GITRD/D2 expressed in responder T cells are good and are downregulated during T cell activation. They may function as a decoy target, impeding GITR activation by GITRL. Thus, the existence of GITR splicing variants and of GITRL reverse signaling together with expression kinetics of GITRL (rarely expressed at high levels for a long time), make it difficult to predict and understand different, sometimes contrasting, results obtained in different experimental settings.

In the following paragraphs we review *in vitro* data explaining how GITR/GITRL modulation affects different aspects of the inflammatory process and the role of antibodies and fusion proteins, which are potentially useful tools in the control of inflammation.

### GITR-GITRL System in Leukocyte Extravasation and Edema

In the above described *in vivo* models there is overwhelming evidence that the GITR/GITRL system is involved in leukocyte extravasation, one of the crucial events of the inflammatory process and shock. However, there is no experimental evidence to fully describe how it happens and it is possible that both GITR and GITRL play a role on endothelial cells. In fact, GITRL is expressed at a high level in endothelial cells and its expression can be modulated by pro-inflammatory stimuli, and GITR is expressed during the inflammatory process.

Adhesion molecules ICAM-1, P-selectin and E-selectin are upregulated in endothelial cells following inflammation, but in the absence of GITR (GITR$^{-/-}$ mice) upregulation is much less evident[23,61]. An obvious explanation is that GITR (expressed on endothelial cells) is triggered by GITRL (expressed on PMNs and monocytes) and participates in upregulation of adhesion molecules. That GITR-activated signals are able to modulate expression of P-selectin and E-selectin is suggested by a study performed on CD3$^{+}$ cells cultured together with an irradiated retinal pigment epithelial (RPE) cell line (ARPE)[58]. In fact, CD3$^{+}$ cells, activated in the presence of a GITRL-transfected ARPE cell line, produced much more P-Selectin and E-Selectin compared to those cultured together with a non-transfected ARPE cell line. The evidence that GITR-Fc fusion protein inhibits extravasation in the described inflammation models suggests a role of GITR in extravasation. Another hypothesis in line with the *in vivo* effect of GITR-Fc fusion protein is that GITRL may function as an adhesion molecule, favoring extravasation of cells that express GITR (such as lymphocytes, PMNs and monocytes). In alternative, since the expression of adhesion molecules is modulated by pro-inflammatory stimuli, such as TNFα[74] and other cytokines, the lack of adhesion molecule upregulation in GITR$^{-/-}$ may be due simply to lower levels of pro-inflammatory stimuli and further studies are needed in this field.

Another feature regulated by endothelial cells is edema, a crucial event in shock and inflammation and due to several mechanisms, including tight junction changes. In some *in vivo* models, GITR$^{-/-}$ mice edema was decreased compared to GITR$^{+/+}$ mice[63,65]. Staining of ZO-1, a marker of tight junction integrity, showed much higher degree of immunostaining disruption in lungs of carrageenan-treated GITR$^{+/+}$ mice compared to carrageenan-treated GITR$^{-/-}$ mice, suggesting a direct or indirect role of the GITR/GITRL system in tight junction integrity[23].

### GITR-GITRL System and Inflammatory Mediators

The early phase of the inflammatory process is characterized by the production of histamine, leukotrienes, platelet-activating factor and COX products, followed by PMN infiltration and production of PMN-derived free radicals and oxidants[75]. Major players in this process are the constitutive isoform of COX (COX-1) and the inducible isoform COX-2. This last isoform is under the regulation of NF-κB and MAP kinase signaling[75,76], and GITR is able to activate both systems. Indeed, there is less COX-2 in the joints of $GITR^{-/-}$ mice in collagen-induced arthritis compared to wild type controls[65]. Moreover, lungs from $GITR^{-/-}$ mice exhibit lower levels of COX-2 expression following carrageenan-induced lung inflammation, and, as expected, $PGE_2$ levels in pleural exudate are reduced[23]. In inflammatory cells from lung tissue, $GITR^{-/-}$ mice expressed lower levels of COX-2 suggesting that macrophages of $GITR^{-/-}$ mice are less activated. This effect may be due to lack of GITR or GITRL triggering. Several studies support the latter hypothesis. In fact, GITRL stimulation by sGITR (the extracellular domain of GITR produced in *E.coli* as a monomer) or GITR-Fc (the extracellular domain of GITR fused with Fc fragment, produced in eukaryotic cells as a dimer) induces COX-2 upregulation and $PGE_2$ production in bone-marrow stromal cells, peritoneal macrophages and RAW 264,7 cell line[29,77]. The same group reports that sGITR inhibits macrophage growth, and since anti-GITR Ab neutralizes this effect, but alone does not affect macrophage growth, they conclude that macrophage cycle-arrest is due to GITRL signaling[73].

Another player in inflammation is NO produced by the inducible isoform nitric oxide synthase (iNOS). NO is important as a toxic defense molecule against infectious organisms. It also regulates the function, growth and death of many immune and inflammatory cell types including macrophages, T lymphocytes, antigen-presenting cells, mast cells, PMNs and NK cells and its target cell specificity depends on its concentration, its chemical reactivity, the vicinity of target cells and the way target cells are programmed to respond. Among the pro-inflammatory effects, NO regulates MMP expression and activity. There are some links between GITR or GITRL triggering, iNOS, and NO production. In CIA and pleurisy models, less iNOS was found in the joints and in the lungs of $GITR^{-/-}$ mice[23,65]. In a series of experiments, Shin et al. demonstrated that GITRL triggering by sGITR induces iNOS synthesis in murine macrophages [78, 79]. Using iNOS inhibitor SMT and NO donor SMP, they demonstrated that there is no correlation between macrophage growth and sGITR induced NO production, so even if there is evidence of NO antiproliferative action, the effects of GITRL triggering do not include inhibition of proliferation[73]. Together, GITR and GITRL promote NO release.

Matrix metalloproteinases (MMPs) appear to regulate cellular behavior through several mechanisms including cell-matrix interactions, extracellular matrix remodeling, angiogenesis, cell growth/apoptosis and the release of bioactive signaling molecules. MMPs are synthesized in response to diverse stimuli including cytokines, growth factors, hormones, and oxidative stress and are involved in the development of several diseases, including inflammatory and vascular diseases. Modulation of GITR/GITRL system causes modulation of some MMPs but data are contrasting. Lee et al. demonstrated that GITRL triggering by sGITR upregulates MMP9 and MMP2 in murine peritoneal macrophages [62]. Accordingly, $CD11b^+$ cells, isolated from virus-infected corneas, increased MMP-9 secretion following anti-GITRL treatment[60]. However, the authors hypothesize that this is due to blocking of GITR/GITRL interaction more than to GITRL triggering. In fact, anti-GITR treatment negatively modulated MMP-9 expression both *in vitro* ($CD11^+$ cells) and *in vivo* (corneal extract of mice with herpes simplex virus infection). Opposite results were obtained by Kim et al., showing that GITR stimulation by anti-GITR Ab induces MMP-9 in mouse and human macrophages from different tissues and *in vitro* monocyte/macrophage cell lines[80]. A possible explanation for the contrasting results is that GITR triggering elicits opposite effects in function of the microenviroment, activation status and type of stimulus. Thus, further studies are needed. Human GITRL triggering (shGITR) induces MMP-13 secretion in fibroblast-like synovial cells and may promote tissue destruction in rheumatoid arthritis[81].

### GITR-GITRL System and Cytokines

#### *Studies on $GITR^{-/-}$ Mice*

As stated before, studies on the inflammatory reaction in GITR$^{-/-}$ mice show decreased inflammation compared to wild type mice. A study on these mice showed that the GITR/GITRL system favors resistance to CIA, proven by less IFNγ, IL-6, TNFα, macrophage inflammatory protein-1 (MIP-1), and MIP-2 secretion[65]. In GITR$^{-/-}$ mice, resistance to TNBS-induced colitis correlated with less IL-12, TNF-α and IL-6 produced by lamina propria mononuclear cells. Moreover, CD4$^+$ lamina propria lymphocytes released less IL-2, and more IL-10 and TGF-β than GITR$^{+/+}$ controls. SAO shock is less aggressive and carrageenan- or bleomycin-induced lung injury is less damaging in GITR$^{-/-}$ mice, and this correlates with less TNFα and IL-1β, compared to GITR$^{+/+}$ controls[23,61,63].

### GITR and/or GITRL Modulation by Fusion Proteins and Antibodies

Several studies demonstrate that anti-GITR antibody (such DTA-1) and recombinant GITRL have agonistic activity on GITR and favor the production of cytokines in various inflammatory cells, both *in vivo* and *in vitro*. In particular, anti-GITR and anti-CD3 Ab treatment induced higher IL-2 and IFNγ levels in T cells, compared to anti-CD3 Ab alone[6]. GITR co-triggering of T cells induces IL-2, IL-4, IFNγ and very strong IL-10 secretion, and this latter seems to counter-regulate enhanced proliferative response[15]. Anti-GITR antibody induced dose-dependent TNFα secretion in mono-macrophage cell lines and increased IL-8, MCP-1 secretion[80]. Cord blood mononuclear T cells (CBMC) show a positive correlation between GITR expression and IL-10 secretion subsequent to allergen exposure[82]. In NKT cells, DTA-1 increased TCR-dependent production of IL-4, IL-10 and IL-13[22]. rGITRL also induced dose-dependent TNFα secretion in Raw 264,7 cells[80]. Administration of anti-GITR Ab during inflammatory reaction induces both Th1 and Th2 type cytokines *in vivo*. Anti-GITR Ab treatment of mice with CIA exacerbated joint inflammation and increased TNF-α, IL-5 and IFNγ production, while anti-GITR Ab treatment of mice with OVA-induced airway inflammation increased IL-2, IL-4, IL-5 and IFNγ[66]. Injection of anti-GITR Ab immediately after HSV-1 viral infection, increased IFNγ secretion by Treg cells[71].

Triggering of GITR is elicited by GITRL expressed on other cells even when they are fixed or irradiated[6,58]. For example, RPE cells which were transfected with GITRL and deadly irradiated, increased T cell-production of a series of pro-inflammatory cytokines as IL-2, IL-6, TNFα, IFNγ, Selectin P and E, and decreases previously high TGFβ levels[58].

*In vitro* GITR triggering induces mainly pro-inflammatory cytokines and promotes inflammation. This is emphasized also by the *in vivo* data, showing that GITR$^{-/-}$ mice have considerably less inflammatory response than GITR$^{+/+}$ controls. However, in some cases, the outcome of GITR triggering can be increased expression of anti-inflammatory cytokines, like IL-4 or IL-10, which presumably tends to limit overextended inflammatory reaction. This apparently contradictory data may suggest that various spectra of induced cytokines have different origins, and different kinetics, contributing to a fine-tuning of the satellite inflammation on proliferation. This advises maximum caution in using antibodies or fusion proteins targeting GITR/GITRL system in therapy. Reverse signalling, and different dynamics of affinity and regulation are highly variable and depend crucially on the kinetics moment, targeted cell type and location.

### GITR/GITRL System and T Cell Activation

Acquired immunity plays a role in some inflammatory reactions. In particular, inflammatory reaction during autoimmune diseases is under the control of T lymphocytes and response to bacteria or viruses is in part due to B and T cells. Despite the several functions of the GITR/GITRL system in innate immunity cells, probably the main role of GITR is played in modulating the effector T cell response. We summarize here how GITR activation modulates activation of effector T cells and function of suppressor T cells (Figure 3). This aspect has recently been discussed in other review papers[9-11].

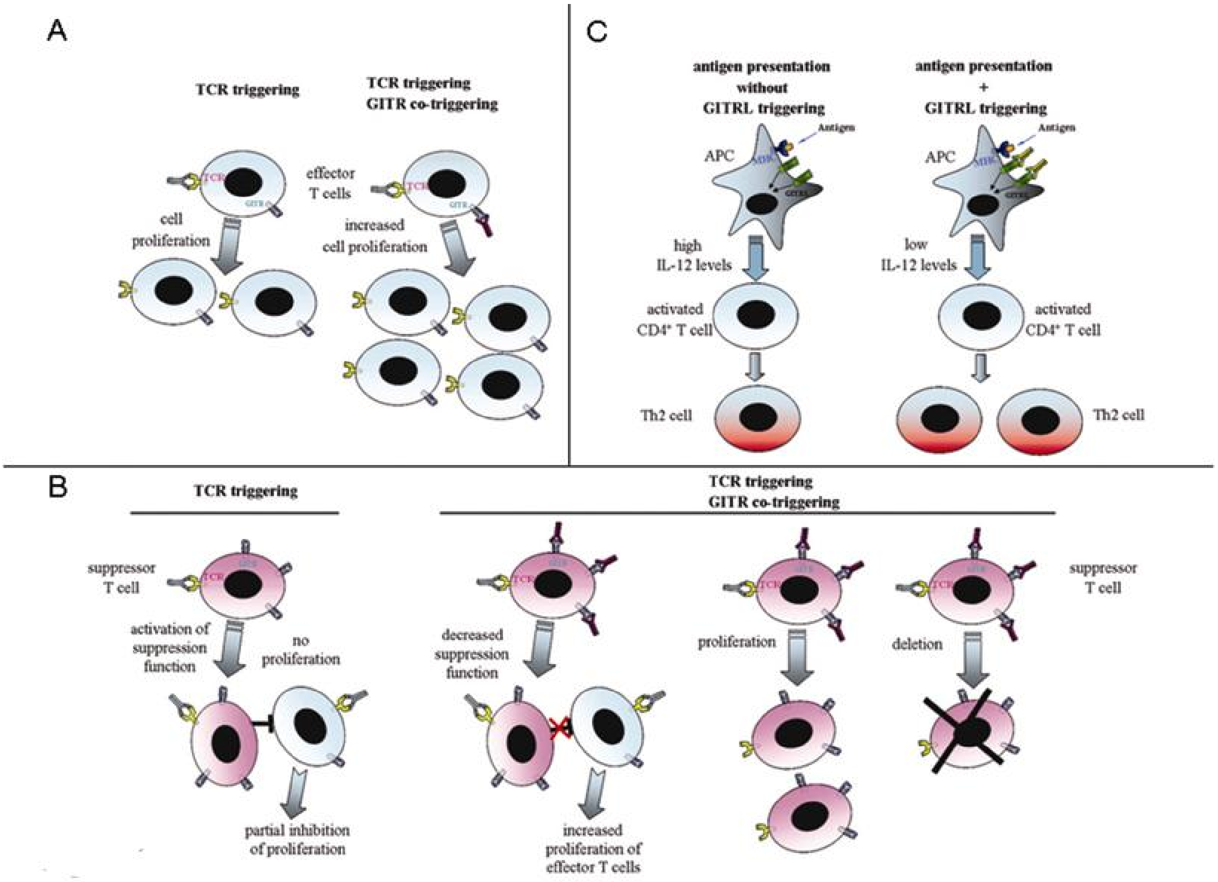

**FIGURE 3.** Role of GITR/GITRL system in modulation of T cell activation

Panel A, co-stimulatory role of GITR; panel B, effect of GITR co-triggering on suppressor T cells (Treg); panel C, switch to Th2 differentiation modulated by GITRL triggering on APC.

### GITR in T Cell Regulation: Co-activating Function on Effector T Lymphocytes

There is overwhelming evidence that modulation of T cell response consequent to GITR triggering derives, first, from co-activation of effector T cells. In fact, GITR co-triggering increases activation and proliferation of TCR-triggered T cells[6,7,15,16,83]. This effect is evident when GITR is triggered by anti-GITR Abs or stimulated by soluble GITRL or GITRL-transfected cells[6,7,14-16]. It is also evident in physiological conditions, as demonstrated by a decreased activation following addition of blocking anti-GITRL Abs to a co-culture of APC (physiologically expressing GITRL) and anti-CD3-triggered T cells[16]. Increased activation is also due to rescue from anti-CD3-induced apoptosis[6]. GITR triggering effects are more evident when TCR is suboptimally activated[7,14](as usually happens for co-accessory molecules) and are evident with lower activation stimuli in $CD4^+$ than in $CD8^+$ cells[16]. In certain experimental conditions, full triggering of TCR and GITR decreases cell proliferation of $CD4^+$ cells[7,15].

Though comparison with other co-stimulatory molecules is hampered by some technical variables, it is believed that the co-stimulatory power of GITR is lower than that of CD28[6,15,19,58] and seems qualitatively different. Studies on total lymph node populations of $GITR^{-/-}$ and $CD28^{-/-}$ mice demonstrated that in the presence of weak CD3 triggering (soluble anti-CD3 in the absence of feeder) and IL-2, the lack of CD28 only in part impaired T cell activation, while the lack of GITR completely abolished T cell activation[16]. This was due, at least in part, to the inability of $GITR^{-/-}$ cells to express the high affinity IL-2R when cocultured with $CD4^+CD25^+$ cells[16]. Studying the effect of retinal pigment epithelial (RPE) cells on T cell proliferation, was demonstrated that GITR triggering abrogated RPE-mediated immunosuppression, while a much smaller effect was seen with CD28 triggering[58], confirming the different effects of CD28 and GITR.

Some studies suggest a different role for GITR in $CD4^+$ and $CD8^+$ cells. During the activation process of $CD4^+CD25^-$ cells, GITR upregulation is mainly dependent on CD28 co-triggering as demonstrated by the greatly increased expression of GITR after CD28-co-triggering and substantial inhibition of GITR upregulation upon activation when physiological CD28 engagement was inhibited by anti-CD80/86 Abs[16,84]. Of note, GITR expression is upregulated by CD28 activation also in the absence of TCR triggering[84], suggesting that a specific signal, not correlated with activation/proliferation, departs from CD28. As a consequence, when CD28 triggering is impeded, the costimulatory effect of GITR-triggering is decreased[16]. Kohm et al. demonstrated that this effect is dependent on CD28-driven IL-2 production[84], while Stephens et al. demonstrated that it is independent of this cytokine[16]. However, the latter used $CD4^+CD25^-$ cells while the former used total $CD4^+$ cells. In conclusion, it seems that, in $CD4^+$ cells, GITR expression and signalling follows CD28 signalling and probably GITR should be regarded as one of the pathways activated by CD28 activation.

In $CD8^+$ T cells the relation GITR/CD28 is somewhat different. In fact, our unpublished studies suggest that in the absence of GITR, $CD8^+$ cells cannot be co-activated by CD28 stimulation when suboptimal doses of anti-CD3 Ab are used while in the absence of CD28, GITR can exert its co-accessory functions (Ronchetti et al., manuscript in preparation). If these findings are confirmed by other experimental models, in $CD8^+$ cells GITR may be a molecule necessary for CD28 costimulatory effects. Even if GITR expression is increased by CD28 triggering, it seems partially independent of CD28 activation[16] (Ronchetti et al., manuscript in preparation). Finally, while full triggering of both GITR and TCR can elicit activation-induced cell death of $CD4^+$ cells, increased TCR stimulation further increases the costimulatory activity of GITR and $CD8^+$ cell activation[16]. These findings may explain why GITR activation potentiates more the response of $CD8^+$ cells than that of $CD4^+$ effector cells in some *in vivo* studies[85, 86].

### GITR in T Cell Regulation: Modulation of The Interplay Treg/Effector Cells

Following GITR triggering, an increased response of the immune system to antigenic stimulation is observed both *in vitro* and *in vivo*. This effect is due not only to costimulation of effector T cells (as discussed above) but also to negative modulation of suppressor T cells (including Treg cells), which are subsets of T cells able to control expansion of effector T cells upon TCR triggering. In 2002, two independent groups working on Treg cells using a different approach demonstrated that GITR activation interferes with the effector/Treg cells interplay[13,19]. Both groups tested Abs directed towards several TNFRSF members with co-accessory function, and demonstrated that anti-GITR Abs were the only ones capable of reverting the suppressor effect of Treg cells[13,19]. Other studies demonstrated the same effect when GITR triggering was exerted by GITRL expressed on APCs[2,6,7,27]. GITR triggering by anti-GITR Ab is also effective in abolishing suppressor activity of other cells such as $CD4^+CD25^-$ T cells present in aged mice[87] or old human donors [88], or retinal pigment epithelial cells[58].

The lower suppressor activity of Treg cells observed in the above mentioned studies can be explained in 2 ways: GITR engagement either inhibits the suppressor activity of Treg cells or makes effector T cells resistant to Treg cell suppression. Since both explanations are well supported by experimental data, it is likely that both contribute to the final effect. The latter hypothesis (effectors resistant to suppression) has recently been proposed by Shevac and Stephens in an "opinion" paper[11] in which they reconsider their own original data[13] in view of the demonstration that GITR triggering is costimulatory for effector T cells[6,7,15,16]. They sustain that "more definitive studies now indicate that signals through GITR costimulate responder T cells and so allow their escape from suppression"[11]. Since the effects of GITR triggering in effector T cells is not impressive and, at best, quantitatively comparable with those obtained with other costimulatory molecules, such as CD28[6,15], this hypothesis would suggest that GITR signaling specifically interferes with the signals activated by Treg cells on effector cells. The data presented by Stephens et al. are in line with this hypothesis[16]. In fact, they demonstrated that total lymph node cells (including $CD4^{+}CD25^{+}$ cells) of $GITR^{-/-}$ mice were unable to proliferate when stimulated by soluble anti-CD3 and IL-2, whereas $CD28^{-/-}$ cells were able, suggesting that the effect of GITR stimulation does not lower only the activation threshold. A similar conclusion was reached by Mahesh et al. investigating the meaning of GITRL expression in human ocular tissue[58]. Expression of GITRL on retinal pigment epithelial (RPE) cells abrogated RPE-mediated immunosuppression of $CD3^{+}$ cells and the effect was independent of Treg cells. It was not a matter of potency in costimulation, but the kind of costimulation, as demonstrated by the very low efficiency of CD28 triggering in abrogating the RPE-mediated immunosuppression vs. a much higher level of costimulation of CD28 in the absence of RPE. In conclusion, these data suggest that in effector T cells GITR triggering activates a pathway (still undisclosed) distinct from that activated by CD28 specifically antagonizing the immunosuppression.

Some *in vitro* and *in vivo* data suggest that GITR stimulation directly affects Treg function. In the first study demonstrating that anti-GITR Ab breaks immunological self-tolerance, Shimizu et al. found that GITR also possesses weak costimulatory activity[19]. Therefore, they used rat responder T cells (on which anti-mGITR Abs do not react) and mouse Treg cells, and demonstrated that the increase in cell proliferation is also due to abrogation of Treg cell activity[19]. Moreover, when Treg cells from $GITR^{+/+}$ mice were cultured together with $CD4^{+}$ effector T cells from $GITR^{-/-}$ mice, anti-GITR Abs (in this experiment effective only on Treg cells), were able to increase the proliferation rate of effector T cells by inhibiting Treg suppressor activity[6]. However, this effect was not observed using mice with another background [16]. In another *in vitro* experiment, the suppressor function of $CD4^{+}CD25^{+}$ T cells on B cells was lost when anti-GITR Abs were added[89]. Some *in vivo* models also confirm direct effects of GITR triggering on Treg cells. Depletion of Treg cells from donor T cells exacerbates GVHD induced by allogenic bone marrow transplantation. T cell-depleted bone marrow cells together with freshly purified effector T and Treg cells were transferred into irradiated mice that received an intraperitoneal injection of anti-GITR antibody[90]. The anti-GITR injected mice died from GVHD while the isotype-injected mice did not. To further demonstrate that survival was due to a direct effect on Treg cells, Treg cells were pre-treated *in vitro* with an anti-GITR antibody, washed and transferred together with the other donor cells in irradiated mice. In this case also, mice developed a lethal GVHD[90]. In fact, transfer of cells depleted of $GITR^{+}$ cells caused severe multi-organ inflammatory disease in Balb/c nude mice, ending in fatal autoimmune myocarditis with anti-myosin antibody secretion, and similarly, transfer of GITR depleted cells from prediabetic NOD mice to NOD-SCID mice accelerated the development of diabetes and induced skeletal muscle myositis and other autoimmune/inflammatory diseases[50]. To test how GITR modulates T cell response during CIA development, spleen cells from $GITR^{-/-}$ or $GITR^{+/+}$ arthritic mice were transferred intraperitoneally into SCID together with collagen[65]. The resulting arthritis was 3.5 fold more severe in $GITR^{+/+}$ transferred mice compared to $GITR^{-/-}$ transferred mice. In this model, GITR derived signals were important in both effector and Treg cells. In fact, when Treg-depleted spleen cells were transferred, CIA was again stronger in $GITR^{+/+}$ transferred mice as compared to $GITR^{-/-}$ transferred mice but at to lesser degree (only 2 fold). Moreover, when Treg-depleted splenocytes from $GITR^{+/+}$ mice were transferred together with $GITR^{+/+}$ or $GITR^{-/-}$ Treg cells, CIA was again stronger in $GITR^{+/+}$ Treg-transferred mice compared to $GITR^{-/-}$ Treg-transferred mice suggesting that physiologic GITR triggering negatively modulates Treg cell activity [65]. A similar result was obtained in the TNBS-colitis model, where, however, the difference between mice transferred with $GITR^{+/+}$ and $GITR^{-/-}$ Treg was not significant due to the already high efficacy of $GITR^{+/+}$ Treg cells[24]. Taken together, the above reported data suggest that, at least in some experimental conditions, GITR triggering also modulates Treg function.

In an attempt to find Treg cell inhibitory signals delivered by GITR, a global gene analysis of anti-CD3 activated Treg cells treated or untreated with anti-GITR Ab was performed[91]. More than 350 genes were transcriptionally modulated 12 hours after GITR triggering, but the full list of genes is not yet available. Granzyme B, a molecule participating in the suppressive/cytotoxic activity of Treg cells, is strongly upregulated in anti-CD3 triggered Treg cells and GITR engagement counters granzyme B upregulation[91], further supporting the hypothesis that GITR negatively modulates Treg cell activity.

GITR costimulation reverses the anergic phenotype of Treg cells after antigen presentation and this effect was correlated to their loss of suppressor function as previously summarized[9], but this may be an oversimplified view. For example, OX40 can modulate Treg function, at least in some experimental conditions, without delivering a costimulatory signal[90]. The pro-proliferative effect of GITR on Treg cells was further confirmed by recent *in vitro* and *in vivo* studies[92]. They also demonstrated that once GITR stimulation has occurred, Treg cells regain their suppressive activity, as previously demonstrated in another experimental setting[19]. The physiological role of GITR for Treg expansion is suggested by a decreased amount of Treg cells in GITR$^{-/-}$ mice[6,16]. The stimulation of Treg cell proliferation by GITR together with the temporary inhibitory effect on Treg function: 1) may limit collateral damage of inflammatory response induced by the exaggerated response to foreign or self antigens, 2) may explain why *in vivo* GITR stimulation does not cause overt autoimmunity[92,93].

Although several studies have claimed that anti-GITR Abs do not lead to depletion of Treg cells[19,70,86,93,94], Shevac and Stephens reported moderate depletion of CD4$^{+}$CD25$^{+}$ cells following an anti-GITR Ab treatment (DTA-1)[11].

In conclusion, GITR triggering may have 4 distinct effects on Treg/effector cell interplay: 1) inhibition for a short time (hours?) of Treg cell suppressor activity by impeding the upregulation of molecules necessary for Treg suppressor activity such as granzyme B, 2) decreased sensitivity of effector T cells to Treg suppression, 3) induction of a partial deletion of Treg cells, 4) promotion of proliferation of functionally active Treg cells, expanding the Treg cell compartment.

#### *GITR in T Cell Regulation: Modulation of The Interplay DC/Treg/effector Cells*

Several studies suggest that professional APCs (i.e. DCs) express GITRL modulating its expression during antigen processing and presentation. Evidence for modulation of DC function by the GITR/GITRL system has been obtained studying *C. albicans* infection of GITR$^{-/-}$ mice[95]. When DCs were cultured in the presence of heat-inactivated C. albicans and GITR$^{+/+}$ or GITR$^{-/-}$ Treg cells, the level of DC-derived IL-12 was lower in DC cocultured with GITR$^{+/+}$ Treg cells. A possible explanation is that GITR (on Treg cells) triggers GITRL (on DCs), modulating DC function. In turn, modulation of DC activity may modulate effector and suppressor T cell activity. Thus, GITRL may modulate immune response not only by triggering GITR on effector and Treg cells, but also by modulating dendritic cell activity through reverse signaling. In the *C. albicans* model the effect of the signaling was pro-inflammatory by favoring Th2 polarization but further studies are needed to fully disclose the effect on DCs.

### HOW THE GITR-GITRL SYSTEM HAS A PLACE IN THE INFLAMMATORY PROCESS: STRUCTURAL FEATURES AND MOLECULAR PATHWAYS

#### GITR Structure and Promoter Region

GITR, like other TNFRSF members, is a type I transmembrane protein formed by a cytoplasmic, a transmembrane and an extracellular domain. Murine and human GITR genes comprise 5 exons[96].

The first 3 exons encode the extracellular domain; exon 4 encodes a small part of the extracellular domain, the transmembrane domain and part of the cytoplasmic domain while exon 5 encodes the cytoplasmic domain.

mGITR is located on chromosome 4 and hGITR on chromosome 1[91,96]. TNFRp75, OX40, CD30, 4-1BB, HVEM and DR3, all belonging to TNFRSF, are similarly located on the murine chromosome 4 and the human chromosome 1, suggesting a common origin and possibly a similar function. However, homology among TNFRSF members is not very high and GITR is not an exception.

Consensus elements for transcription factors involved in the inflammatory response were identified in the 5’ flanking region of the GITR gene[96](Bianchini, unpublished). Several consensus elements involved in the inflammatory process are present, including NF-κB, STAT5, SRF, LEF1, NF-AT1, NF-IL6, IFR4 and TFE3. They are crucial both for activation of T and innate immunity cells, further suggesting the role of GITR in the inflammatory process.

The above-mentioned studies demonstrate GITR expression in skin and bone. GITR expression in these tissues is also suggested by the presence of TFE3 and LEF1 (skin), and LEF1, STAT5, OCT1P and GKLF.1 (bone). Some elements (e.g. MYT1 and PTX1) promoting gene expression in neurons have been found, confirming the potential expression of GITR in the central nervous system, as suggested by GITR presence in the brain. Finally, two highly significant binding sites for MyoD and one for myogenin have also been found, suggesting that GITR is involved in muscle development[96]. Although the weak expression of GITR in neuron and muscle (Table 2) the specific expression of GITR in different conditions and its role in these tissues deserves further investigation.

### The Extracellular Domain of GITR

TNFRSF members are characterized by cysteine-rich domains in their extracellular portion. Cysteines form disulfide bridges, which contribute to structurally defined binding sites for the specific ligand[97]. A canonical cysteine-rich domain (CRD) contains 6 cysteines (numbered 1 to 6) which form 3 disulfide bonds (C1 with C2, C3 with C5 and C4 with C6)[97,98].

**Table 3.** Motifs present in the extracellular domain of TNFRSF members

| Name of the motif | Amino acid length | Motif and position of the cysteines |
|---|---|---|
| **CRD1** | 33-38 | **C**-x(4,6)-Y-x(5,8)-**C**-**C**-x(2)-**C**-x(2)-G-x(6)-**C**-x(6,8)-**C**<br>1 2 3 4 5 6 |
| **CRD2/4** | 36-43 | **C**-x(0,1)-[EDP]-x(0,1)-[GSD]-x(0,2)-[FY]-x(5,10)-**C**-x(2)-**C**-x(2,3)-**C**-x(8,11)-**C**-x(4,5)-[NDKR]-x(1,2)-**C**<br>1 2 3 4 5 6 |
| **CRD3** | 34-42 | **C**-x(5,6)-[**C**Q]-x(4,8)-[**C**HST]-x(2)-**C**-x(1,3)-[**C**HR]-x(1,3)-**C**-x(4,5)-[VLI]-x(2,4)-[**C**AG]-x(4)-[ND]-x(2)-**C**<br>1 2 3 4 5 6 7 8 |
| **CRD4** | 38-42 | **C**-x(2)-G-x(1,2)-[FY]-x(6,9)-**C**-x(2)-[**C**WH]-x(2)-**C**-x(9,13)-[**C**GA]-x(4)-[DN]-x(2)-**C**<br>1 2 3 4 5 6 |

Although GITR belongs to TNFRSF, a canonically defined CRD according to BLAST utility (NCBI) is not present in GITR. To better define CRD, we compared the primary structure of TNFRSF members extracellular domain, and identified four different motifs based on cysteine position, conserved amino acidic residues and the spaces between (Table 3). These motifs, when present, are located in different positions of the extracellular domain and consequently, we named them CRD1 (next to signal peptide), CRD2/4, CRD3 and CRD4 (next to transmembrane). CRD1 and CRD2/4 motifs have 6 cysteines and are variants included in the canonical CRD. On the contrary, CRD3 and CRD4 motifs were not described so far as motifs characterizing the extracellular domain of TNFRSF

members despite being observed in more than 35% and 50% respectively of members belonging to this family (not shown).

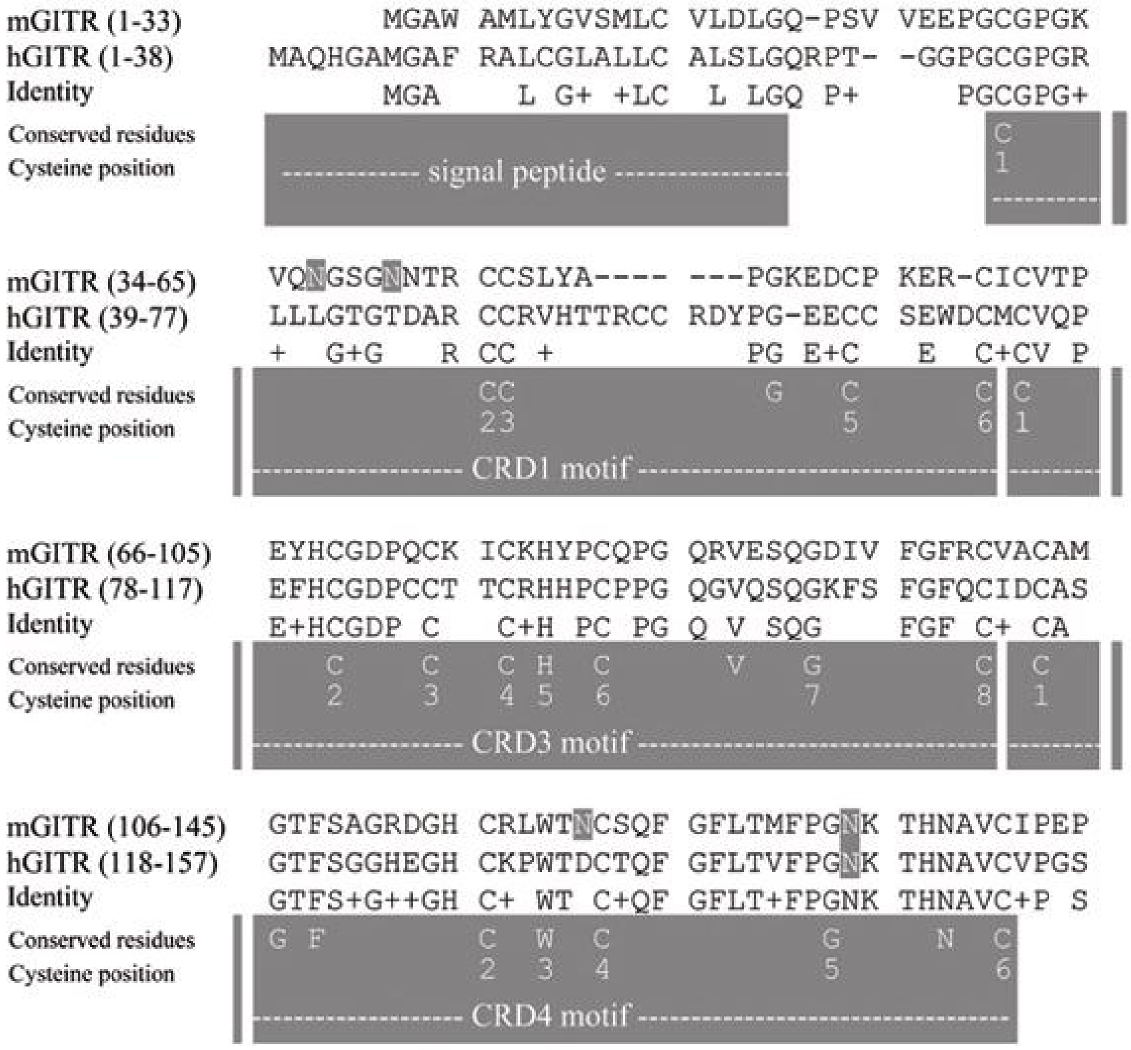

**FIGURE 4.** Homology between the extracellular domains of mouse and human GITR.

Comparison between the extracellular domains of mGITR and hGITR. The amino acid position is reported between brackets. The Asparagine in white on gray background represents potential glycosylation sites. In the identity line, amino acid residues with similar function (I, V, M, L; H, R, K; E, D, N, Q; S, T; A, G; Y, F) present in both sequences are indicated by +. In the conserved residues line, the amino acid residues matching the respective CRD (see Table 3) are reported. Cysteine position in the CRD (or the position of the amino acid residue substituting the cysteine residue) is also reported.

The CDR3 motif (Table 3) contains from 4 to 8 cysteines forming disulfide bonds in a way different from that of the canonical CRD: C1 with C4, C2 (if present) with C3 (if present), C5 (if present) with C7 (if present) and C6 with C8, as demonstrated by Banner et al. in the crystallographic study of TNFR[97]. When cysteines are absent, only some amino acid residues can replace them. The CRD4 motif (Table 3) usually contains 4 cysteines, cysteine 3 being replaced by tryptophan or histidine residues and cysteine 5 by alanine or glycine residues.

GITR contains a badly conserved CRD1, a fairly well conserved CRD3 and a perfectly matched CRD4 (Figure 4). In contrast, it lacks CRD2/4. Atypical CRD1 in GITR lacks the cysteine residue C4 and the tyrosine residue located after C1. Moreover, the amino acid residues between C3 and C5 are too few in mGITR and too many in human hGITR, to form a disulfide bond. Therefore, the CRD1 motif in GITR might contain just 1 disulfide bridge and may not represent a structurally defined CRD. Low conservation of the sequences representing this motif (51% similarity between mGITR and hGITR, Figure 4) in mGITR compared to hGITR suggests that CRD1 has little functional meaning in GITR. CRD3 is quite well represented in both mGITR and hGITR. The only missing amino acid residue is either the asparagine residue or the glutamic acid residue located near C8. The motif is characterized by 6 cysteine residues while C5 (H is instead present) and C7 (G is instead present) are lacking. CRD4 is perfectly represented in both mGITR and hGITR. The crucial meaning of CRD3 and CRD4 in GITR is further supported by the high similarity of mGITR compared to hGITR in these domains (61% and 75% similarity, respectively) and by the conservation of CRD3 and CRD4 in other species (*Bos taurus, Canis familaris, Macaca mulatta,* and *Pan troglodytes*) with a 60-65% similarity (CRD3) and 65-75% similarity (CRD4). GITR does not show a high homology towards other TNFRSF members in the extracellular domain, though. This explains why GITRL is extremely selective for GITR[8].

Reports are not available on the role of the CRDs of GITR in GITRL binding. Studies on other TNFRSF members proved to be difficult to predict the role of the different CRDs. For example, in Fas (TNFRSF6) the domains corresponding to CRD2/4 and to CRD3 play major roles in ligand binding. In TNFRI the domains corresponding to CRD1, CRD2/4 and CRD3 play a role, and in NGFR the domains corresponding to CRD3 and CRD4 are crucial[99,100]. The role of the different CRDs is interesting not only from a theoretical point of view but also for understanding the role of GITRD and GITRD2, soluble products of GITR gene, potential competitors for GITRL binding.

Both murine and human GITR have potential glycosylation sites: mGITR has 4 sites, whereas hGITR has only one that is conserved with respect to mGITR (see Figure 4). Western blot experiments indirectly confirmed that mGITR is glycosylated, since molecular weight of mature mGITR, calculated on the basis of amino acid composition, is 23.3 kDa, while experimental molecular weight ranges from 35 to 40 kDa, depending on the cell population tested [101].

### The Cytoplasmic Domain of GITR

The cytoplasmic domain of mGITR and hGITR is respectively 52 and 53 amino acid residues long, and shows a good homology with the cytoplasmic domains of OX40, 4-1BB and CD27 (similarity between 45 and 50%)(Figure 5)[96]. The homologies span the complete cytoplasmic domain but are centered in 2 segments: domain 1, the sequence next to the –COOH terminus of transmembrane region, and domain 2, close to the –COOH terminus of the proteins (Figure 5). Interestingly, domains 1 and 2 are coded by different exons[96].

Domain 1 is present in mGITR, mOX40, hOX40, m4-1BB, h4-1BB, mCD27 and hCD27, is characterized by 3 basic residues and is described by the motif [KR]-[KR]-x(0,2)-[KHR]-x(0,2)-[PY]. A similar motif ([KR]-[KR]-x(0,3)-[KHR]-x(1,5)-P) is also found in TNFRp75, CD40 (TNFRSF5) and HVEM (both murine and human). Figure 5 shows that hGITR lacks 2 (hGITR) or 1 (hGITR variant) basic residues, which are deleted compared to the rest of the family members, but whether this lack has functional implications (activation of partially different pathways by mGITR and hGITR triggering) remains to be determined.

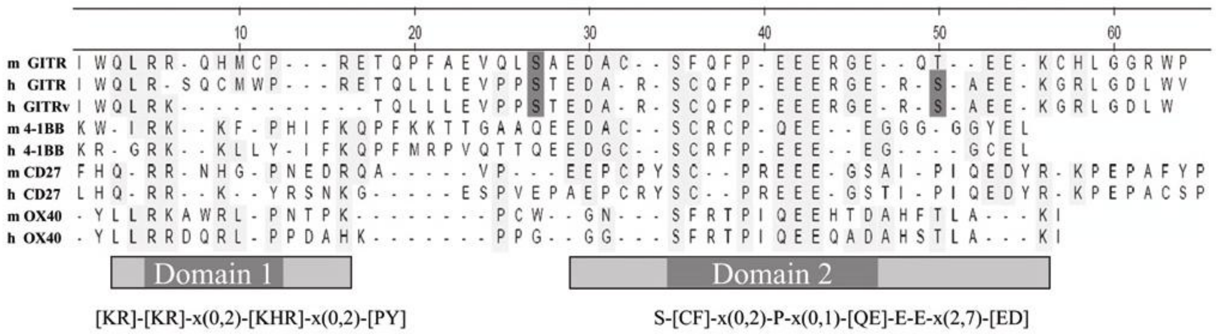

**FIGURE 5.** Homology among the cytoplasmic domain of GITR and the other human and mouse TNFRSF members of GITR subfamily.

Comparison between all the cytoplasmic domains of mouse and human GITR (mGITR, from amino acid 177 to amino acid 228; hGITR, from amino acid 189 to amino acid 241; hGITRv, a splicing variant of hGITR, called variant 3, from amino acid 189 to amino acid 233), 4-1BB (m4-1BB, from amino acid 212 to amino acid 256; h4-1BB, from amino acid 214 to amino acid 255), CD27 (mCD27, from amino acid 202 to amino acid 250; hCD27, from amino acid 211 to amino acid 260) and OX40 (mOX40, from amino acid 234 to amino acid 272; hOX40, from amino acid 238 to amino acid 277). Amino acid residues identical or with similar function (I, V, M, L; H, R, K; E, D, N, Q; S, T; A, G; Y, F) present in more than 50% of the sequences are shaded. The serine in dark gray background are potential Casein Kinase II phosphorylation (CKII) sites.

Domain 2, the longest, is characterized by several acid residues (up to 8 in GITR, both human and murine) and a few basic residues. The more conserved portion is described by the motif S-[CF]-x(0,2)-P-x(0,1)-[QE]-E-E-x(2,7)-[ED], which is present in GITR, OX40, 4-1BB and CD27 (all involved in the inflammation/immune response, promoting activation and survival of immune cells). In the cytoplasmic domain of CD40, which is involved in B cell activation and survival, 2 similar motifs are present: T-x(2)-P-x(1)-Q-E-x(10)-E and T-x(3)-C-x(1)-P-x(2)-Q-E-D-x(2)-E[101]. Domain 2 therefore can be called the “life domain” as opposed to the death domain in other TNFRSF members, whose triggering activates apoptosis.

Motif 1 and motif 2 are shared by several (but not all) TNFRSF members whose genes are present in the same chromosomes where GITR is found. This may be a consequence of chromosome duplication. However, as CD40 and CD27 are not located in the same chromosomes as GITR, a functional convergence should be hypothesized rather than an evolutionary consequence. In conclusion, the good homology in the cytoplasmic domain between GITR, CD27, OX40 and 4-1BB and a quite good homology with CD40, together with the similarity in function, lead us to define a new subfamily of TNFRSF[1,96].

### *Alternatively Spliced Products of GITR*

Several members of TNFRSF are characterized by splicing variants[102,103] and both murine and human GITR genes originate alternatively spliced products. Besides mGITR, the mGITR gene produces 2 more alternatively spliced products (mGITRB and mGITRC) (Figure 6)[104], which share an identical extracellular domain and a transmembrane domain with mGITR. The –$NH_2$ terminus of the cytoplasmic domain (containing the above mentioned domain 1) is identical also in mGITR, mGITRB and mGITRC, but the –COOH terminal cytoplasmic domain is completely different. In fact, in GITRB, 11 base pairs of intron 4 are present, thus changing the open reading frame of exon 5. In GITRC, the short intron 4 (67 bp) is unspliced and the open reading frame of exon 5 is different from both mGITR and mGITRB. In T lymphocytes both splicing variants are expressed at low levels compared to mGITR, with GITRB being the less expressed. However, performing a library screening of a $CD4^+$ T cell hybridoma, we found that GITRB was the predominant GITR gene splice variant, indicating that it may be expressed at high levels in a subpopulation of $CD4^+$ cells[104]. The cytoplasmic domain of mGITRB contains, among others, a potential binding domain for $p56^{lck}$[104].

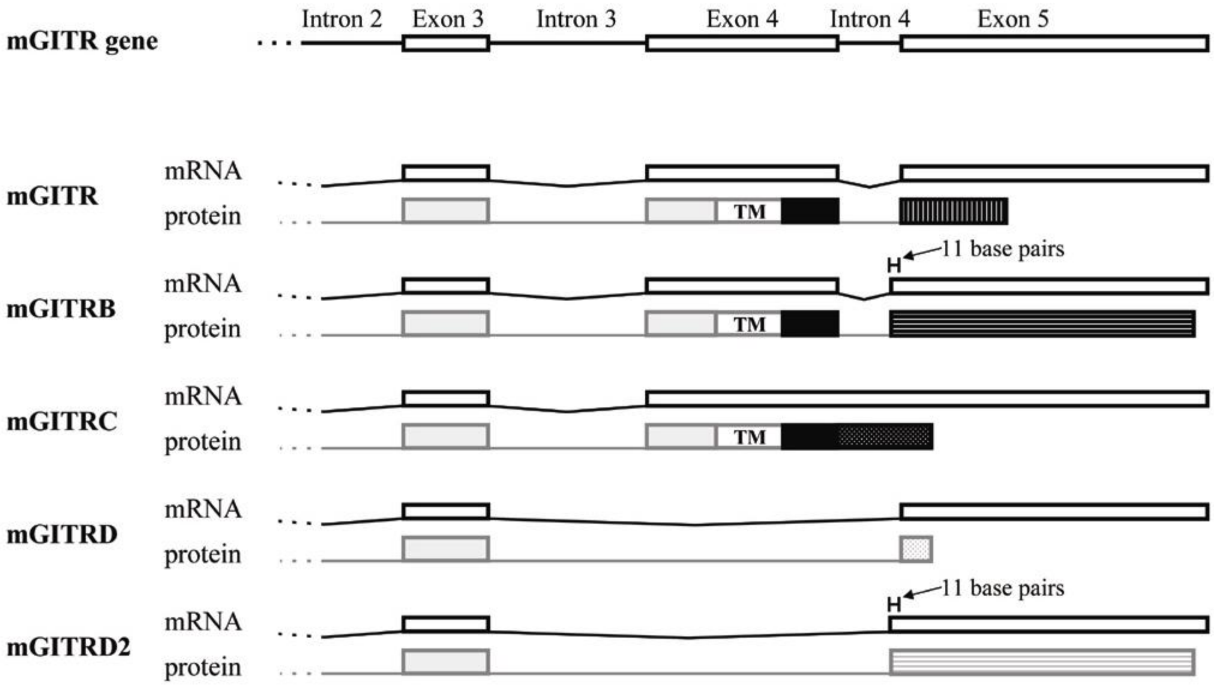

**FIGURE 6**. Murine GITR splicing variants.

Exonic structure of mGITR and comparison among its alternative splicings (exon 1 and 2 are not shown because not involved in alternative splicing). The putative proteins are characterized by an extracellular domain (gray boxes), a transmembrane domain (empty boxes labeled with TM) and a cytoplasmic domain (when present, black boxes and boxes with a black foreground). Exon 5 has 3 different open reading frames, indicated by the vertical line pattern (mGITR), the horizontal line pattern (mGITRB and mGITRD2) and the pointed pattern (mGITRC and mGITRD).

We also cloned 2 soluble spliced products of the GITR gene: mGITRD[104] and mGITRD2 (unpublished, GenBank accession number AF241229)(Figure 6). mGITRD and mGITRD2 mRNAs skip the exon 4, and thus these splicing variants lack the transmembrane domains and, as demonstrated experimentally, are soluble proteins (Nocentini et al., unpublished). As they contain the entire CRD1 and CRD3 motifs in mGITR together with a small part of the CRD4 motif (including Cysteine 1 and Cysteine 2) they may bind GITRL. Moreover, in peripheral T cell populations expression of mGITRD/D2 and mGITR are similar but mGITRD/D2 expression decreases following T cell activation[104]. All together, these observations suggest that these soluble proteins may function as decoy targets for GITR and interfere with GITR-GITRL interaction.

At present, only 3 splice variants of hGITR were described. The full length GITR (ortholog to mGITR) is called variant 1 and was originally cloned by Gurney et al.[4]. Variant 3, originally cloned by Kwon (we call hGITRv, Figure 5), lacks the 21 bp present at the 3’ end of exon 4[3], and consequently, 7 amino acids in the cytoplasmic domain, located inside motif 1. It has, however, an identical open reading frame of exon 5. Variant 2 (also called hGITRD) lacks exon 4 and thus the transmembrane domain (GenBank number AF241229 and NM_148901, Nocentini et al., unpublished). In peripheral blood lymphocytes, hGITRD is expressed at very low levels. No other data are available on the expression of human variants.

None of the functional data we have for GITR, deals with GITR splicing variants, although knowledge of their tissue distribution and their function might be useful for understanding appearantly contradictory results.

### GITR Triggering: The Molecular Basis of Proinflammatory Effects

#### *TRAF Pathways Leading to NF-κB Activation*

There is clear evidence that GITR binds TRAF2, TRAF1 and TRAF3 but does not bind TRAF6[3,4,83]. Recently, Haurer et al. demonstrated that GITR binds TRAF5 but the binding is relatively weak compared to CD40[105]. TRAFs bind TNFRSF as a trimer through the amino-terminal RING and zinc-finger motifs[106]. Following TRAF binding, several pathways are activated, including MAP-kinases signaling, and finally, activation of NF-κB and AP-1 family transcription factors, deeply involved in the inflammation process. Kwon et al. demonstrated that overexpression of hGITR alone induces NF-κB activation, that is further increased when TRAF2 is coexpressed. Coexpression of TRAF1 or TRAF3 downregulates activation of NF-κB below the levels of GITR overexpression alone. Overexpression of dominant negative TRAF2, lacking RING and zinc finger motifs, abolished the hGITR induced effects. A similar result was obtained by overexpression of a dominant-negative NF-κB inducing kinase (NIK), a transduction factor downstream TRAF2 in the NF-κB signaling pathway. In conclusion, GITR activates the NF-κB pathway by TRAF2/NIK, and downregulates it by TRAF3 and TRAF1[3].

Several other TNFRSF members (including 4-1BB, CD27, OX40, CD40) bind TRAFs and, frequently, TRAF binding involves the P-x-[QE]-E consensus, which is present in the above defined motif 2 (Figure 5)[106,107]. Ji et al worked with mGITR mutants lacking the 13 (GITR-Δ215) or the 22 (GITR-Δ206) –COOH terminal amino acids. They demonstrated that GITR-Δ206 was unable to activate NF-κB upon sGITRL binding. On the contrary, GITR-Δ215 responds to sGITRL even more than wild type GITR indicating that the amino acid residues FQFPEEERGE (including P-x-[QE]-E consensus) are necessary for activation of NF-κB and that the –COOH terminal region represents a negative regulatory element[27].

Surprisingly, Esparza et al. demonstrated that mGITR coexpressed with TRAF2 reduces significantly instead of increasing NF-κB activation, as in the case of hGITR. Of note, hGITR used by Kwon was hGITRv, lacking 7 amino acidic residues present in domain 1, which do not represent TRAF binding motif but may modulate TRAF function. Other reasons, potentially explaining the different effects of GITR-induced TRAF2 activation, are a different GITR-activated transduction pathway in humans and mice or different experimental settings. mGITR activation also seems to induce a new intracellular localization of TRAF2 from cytoplasm to plasma membrane. TRAF4 antagonizes all TRAF2 inhibitory effects on GITR-induced NF-κB activation, relocating TRAF2 inside the cell. However, TRAF4, not binding GITR directly, has been supposed to use an adaptor protein to interact with GITR[108]. Since TRAF4 has two nuclear localization sequences, GITR-mediated NF-κB activation may be due to TRAF4 shuttling between cytoplasm and nucleus[10].

There are two pathways of NF-κB activation: 1) a canonical pathway via IKKβ resulting in the degradation of IκBα and nuclear translocation of p50/RelA heteromers requiring the protein IKKγ (NEMO), 2) a non-canonical pathway signaled by NIK (NF-κB-Inducing Kinase), a kinase activating IKKα operating independently of IKKγ; IKKα activation causes NF-$\kappa B_2$ (p100) degradation and nuclear translocation of p52/RelB. For example, CD40 has two signaling options for NF-κB activation: via TRAF6, canonical, and via TRAF2/5-NIK, non-canonical. TRAF3 blocks this latter activation[105]. To elucidate the other possible transduction pathways activated by GITR stimulation and related to the non-canonical NF-κB pathway, the effect of GITR activation on TRAF5 was analyzed [109]. GITR triggering on TRAF5-deficient T cells elicits an inhibitory effect on NF-κB and MAPK p38 and ERK, while JNK (c-Jun N-terminal kinase) is less affected. However, while overexpression of TRAF2 and TRAF4 was not sufficient to activate the NF-κB pathway, expression of TRAF5 was. TRAF5 deficiency provokes a downregulation of anti-GITR-Ab-induced enhancement of antigen-depending T cell proliferation consistent with reduced NF-κB, p38 and ERK, which is not verified following CD28 triggering [109]. These studies suggest that TRAF5 is the main transduction protein able to activate NF-κB following GITR triggering. However, lack of TRAF5 does not abolish completely the GITR-dependent NF-κB activation, so there must also be a TRAF5-independent mechanism involved, like the one discussed above[10]. The role of NIK, a component of non-canonical NF-κB signaling, will be treated below.

Activation of NF-κB has been studied in effector and suppressor T cells. Studies on $CD4^+$ and $CD8^+$ peripheral T lymphocytes from $GITR^{+/+}$ and $GITR^{-/-}$ mice demonstrated the induction of activation of the NF-κB pathway following TCR and GITR co-triggering. In particular, when $CD4^+CD25^-$ T cells from $GITR^{+/+}$ mice were triggered with anti-CD3 and anti-GITR, p42 MAPK phosphorilation was higher than in T cells from $GITR^{-/-}$ mice, indicating that GITR is involved in the MAPK pathway activation[6]. Consequently, NF-κB is more activated in co-triggered $GITR^{+/+}$ mice compared to $GITR^{-/-}$ mice. A similar result was obtained by Kanamaru et al. working with anti-GITR antibodies. In fact, nuclear fractions of p50, p65 and c-Rel (3-fold) are increased after (suboptimal) anti-CD3 + anti-GITR mAb treatment compared to anti-CD3 treatment alone[15].

### Siva Pathway and Cell Death

GITR has no death domain in its structure, unlike Fas or TNFR1. Other structural homologues like CD40, 4-1BB, OX40 and CD27 lack death domains. The last member can induce apoptosis by binding a protein that has a death domain homology region in its central region, called Siva[101,110]. Several studies show that Siva is upregulated in various pathological conditions such as acute ischemic injury, Coxsackie virus infection, or anticancer treatment such as the TIP30 metastasis suppressor, which inhibits metastasis of the small cell lung carcinoma by predisposing cells to apoptosis[111]. Siva-induced apoptosis is caspase-dependent, with caspase 8 activated upstream of caspase 9 and 3[111]. Moreover, Siva directly activates a pro-apoptotic Bcl-2 family member (Bid) and inhibits anti-apoptotic Bcl-2 family members (Bcl-2 and Bcl-xL).

Spinicelli et al. demonstrated by co-immunoprecipitation that GITR binds Siva, and that overexpression of GITR and Siva leads to apoptosis[101]. It is generally accepted that overexpression of TNFR members promote low levels of activation, so it was assumed that co-transfection mimics GITR triggering. In addition, an increased level of apoptosis was observed after anti-mGITR Ab treatment[101]. In mGITR, SFQFPEEE (position 205-212) is the sequence responsible for Siva binding, with the PEEE sequence playing an important role[101]. In hGITR, the QFPEEE sequence (position 219-224) is conserved providing further evidence that it has a functional role. Since CD27 and OX40 also bind Siva, sequence alignment supports the conclusion that P-[IE]-[QE]-E is the main binding motif for Siva[101]. The domain formed by the above-mentioned amino acids is also capable of TRAF binding. Thus, it is possible that, depending on the functional status of the cells, triggering of these receptors may lead to different effects. Indeed, in both mGITR and hGITR, potential Casein Kinase II phosphorilation (CKII) sites are present in the Serine at position 199 (S199, mGITR), position 211 (S211, hGITR) and position 229 (S229, hGITR) (Figure 5). S199 in mGITR is conserved, corresponding to S211 in hGITR. The Serine 199 (mGITR) is also necessary for Siva binding[101] and preliminary experiments suggest that S199 phosphorilation modulates TRAF2 and Siva binding in opposite ways (Nocentini et al., unpublished). Lu et al. studied $CD4^+$ T cells from NIK deficient mice, a transduction factor mainly belonging to the non-canonical signaling able to activate NF-κB[112]. Effector T cells of $NIK^{-/-}$ mice were normal for both activity and proliferation after TCR/GITR co-triggering. On the contrary, Treg cells were normally suppressive but after TCR/GITR co-triggering they proliferated much more than in wild type mice, suggesting the involvement of NIK in GITR costimulation of Treg cells. Since Siva binds to and inhibits NIK[113], Lu proposed that GITR-dependent activation of Siva is possible only when NIK is present[112]. Thus, NIK and Siva may function as a negative control of GITR co-triggered proliferation.

As previously discussed, NIK is related to TRAFs. In particular, TRAF3 is a negative controller of NIK and promotes NIK degradation, while TRAF5 is an inducer of NIK activation and counteracts TRAF3-dependent NIK degradation[114]. Therefore, NIK levels are dependent on TRAF3/TRAF5 levels and activation, which may be different in Treg and effector T cells, explaining the abnormal response of Treg cells to GITR co-triggering. Moreover, Siva is required for TCR-induced apoptosis. In fact, Siva deficiency leads to resistance to anti-CD3, but not to Fas-induced apoptosis and in Siva-deficient cells canonical and non-canonical pathways of NF-κB are significantly increased with high levels of nuclear p65 and Rel B, respectively[115]. In conclusion, Siva, NIK, TRAF3 and TRAF5 levels seem crucial for the final effect of GITR triggering, including NF-κB activation.

#### PRMT1 Pathway

The cytoplasmic domain of GITR (next to transmembrane domain) is also quite similar to a portion of BTG2 (Figure 7), a cytoplasmic protein belonging to the BTG/TOB family. Several members of this family play a role in cell cycle negative control and differentiation[116]. BTG1 and BTG2 interact with the protein arginine N-methyltransferase (PRMT1) and modulate its activity positively[117]. PRMT1, the enzyme that catalyses most of the type-I methylation reactions, is involved in protein trafficking, signal transduction and transcriptional regulation such as transcriptional activation promoted by p53[118]. Deletion studies have demonstrated that the DGSICVLYEE peptide is necessary for BTG1/2-PRMT1 binding (see PRMT1 binding domain of Figure 7). However, this peptide is not sufficient, suggesting that other portion of BTG1/2 are important excluding portion from 125 to the –COOH end of the proteins, which is unnecessary[119]. The high similarity between GITR and BTG2, shown in Figure 7, is concentrated on the PRMT1 binding domain and in a portion next to -$NH_2$ terminus of PRMT1 binding domain. Therefore, the above-mentioned study and the GITR/BTG2 alignment suggest that GITR could also bind PRMT1. Indeed, *in vitro* studies indicate the cytoplasmic region of mGITR binds PRMT1 but the mGITR –COOH deletion mutant (deleted starting from Alanine 200) does not bind PRMT1 (Nocentini et al., unpublished data).

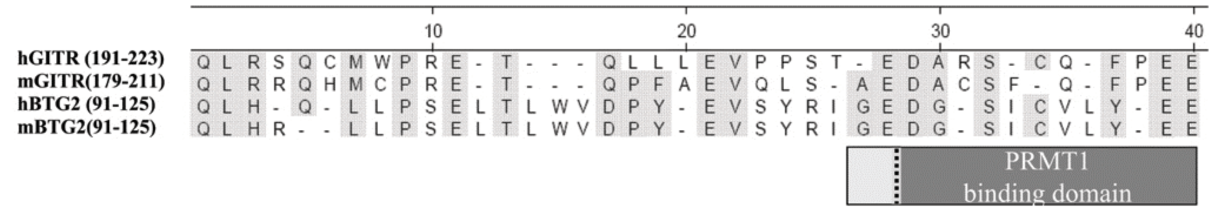

**FIGURE 7**. Homology beetween BTG2 e GITR

Comparison between a portion of the cytoplasmic domain of mouse and human GITR and a portion of mouse and human BTG2 (amino acid position is indicated between brackets). Identical amino acid residues or those with similar function (I, V, M, L; H, R, K; E, D, N, Q; S, T; A, G; Y, F) present in at least 3 sequences are shaded. The potential PRMT1 binding motif is indicated.

PRMT1 deficiency is lethal, having a crucial role in gene transcription modulation and regulating protein interaction[120]. PRMT1 is located in both cytoplasm and nucleus with a preference for cytoplasm and its localization is modulated by the concentration of its substrates and products[120]. Moreover, it is highly expressed in activated T helper cells[121]. One target of PRMT1 is NF-κB, whose activity is stimulated 10 fold by PRMT1 in transfection experiments[122]. For example, shear stress on endothelial cell lines HUVEC and ECL-305 induces NF-κB activation and PRMT1 upregulation[123]. Another target of PRMT1 is NF-AT interacting protein (NIP45), a protein bound by TRAF2 and TRAF5 and negatively modulated by them[124,125]. NIP45 modulates NF-AT activity and upregulates IFNγ and IL-4 gene transcription[121].

Although the functional significance of the GITR/PRMT1 interaction is presently unknown, GITR-PRMT1 binding suggests that the role of GITR in modulating the inflammatory/immune reaction may also be due to PRMT1 activation.

### GITRL Structure and Signaling

GITR ligand is a member of the TNF superfamily (TNFSF)[3,4]. Murine GITR ligand (mGTRL) was only discovered in 2003[2,5-7], while its human ortholog was discovered simultaneously with its receptor in 1999[3,4]. The encoded protein is a type II transmembrane protein of 173 amino acids and an apparent molecular weight of 20 KDa. This protein has 51% homology with its human ortholog, and is divided into an N-terminal cytoplasmic domain of 21 amino acids, a transmembrane and a –COOH terminal extracellular domain of 129 amino acid –COOH terminal part[2]. mGITRL gene is 9.3 Kb long, incorporating 3 exons: exon 1 of 135 bp, exon 2 of 34 bp and exon 3 of 353 bp[7].

Nuclear factor NF-1 is responsible for LPS-induced GITRL upregulation, which acts specifically on the GITRL promoter (consensus TTGGCCTGGTGCCAC)[7].

#### *The Extracellular Domain of GITRL*

Analyzing GITRL using PFAM motif databank, there is homology with the TNF family starting from amino acid 61 to amino acid 166 (PFAM PF00229 interpro ipr006052), which makes it structurally matched to the TNF superfamily. Based on its structure, as is the case of other members of TNF, the trimer-formation is highly probable for the ability to stimulate GITR receptor. In the GITRL extracellular domain there is a hypothetical glycosylation site on Asn 74. Indeed, experimental molecular weight (western blot) is 25-28 kDa, that may be explained by different states of glycosylation[5]. Mahesh et al. found a soluble form of GITRL, potentially due to shedding[58]. Indeed, dedicated softwares give us various cleavage sites (not shown), but so far no experimental evidence of enzymatic cleavage has been published.

#### *The Cytoplasmic Domain of GITRL*

The cytoplasmic domain of GITRL shows a high homology with OX40L[95] (Figure 8). In both mouse and human, the GITRL gene is next to OX40L gene, suggesting that GITRL and OX40L derive from the duplication of an ancestral gene.

It has been hypothesized that GITRL is able to activate an intracellular signal following GITR binding[29,73,78,79]. This possibility is further suggested by the high homology in the cytoplasmic domain among mouse, human and other mammals (including *Bos taurus, Canis familaris, Macaca mulatta* and *Pan troglodytes*), so it is possible to find a conserved defined motif: (E-x-M-P-L-x(2)-S-x(2)-Q-x-A-x-R-x(2)-K-x-W-L). This motif is peculiar to GITRL gene. There is no evidence of domains for kinase or other enzymatic activities and there is no experimental data regarding binding of transduction proteins. The only evidence for a potential transductional role of GITRL is the presence of a potential phosphorilation site on Ser10.

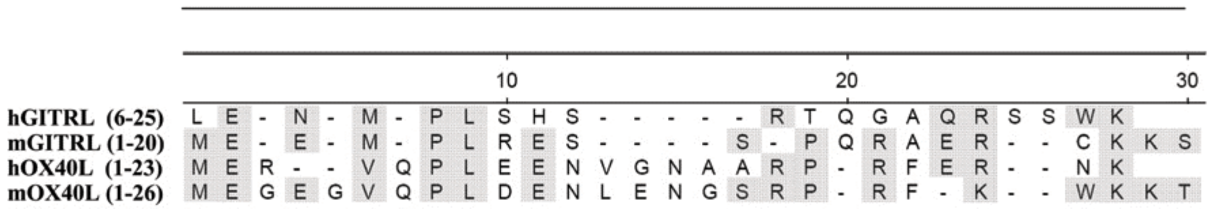

**FIGURE 8.** Homology between the cytoplasmic domain of GITRL and OX40L

Comparison between the cytoplasmic domain of GITRL and OX40L (amino acid position is indicated between brackets). Identical amino acid residues or those with similar function (I, V, M, L; H, R, K; E, D, N, Q; S, T; A, G; Y, F) present in at least 2 sequences belonging to a different gene are shaded. The serine amino acidic residues in dark gray background are potential phosphorilation sites.

#### *Splicing Variants of GITRL*

mGITRL mRNA has a potential RNA destabilization signal consisting of AU-rich sequences near the 3' end, in the UTR. An isoform mRNA, without this destabilization signal, was revealed in IL-10-treated bone marrow derived DC that express high levels of mGITRL mRNA. This alternative splicing derives from the substitution of a part of exon 3 (UTR region) with exon 4. Thus, mGITRL mRNA levels might be controlled also by post-transcriptional regulation[7]. No alternative splicings giving different GITRL proteins were cloned so far.

### CONCLUDING REMARKS

GITR functions as an activating molecule in several cells of innate and acquired immunity. It is triggered by GITRL that can also deliver cytoplasmic signals, most of which have a pro-inflammatory meaning. Moreover, the GITR/GITRL system seems to play a crucial role in the extravasation process. Thus, it is not surprising that GITR/GITRL system has pro-inflammatory role in several *in vivo* models including those of shock, acute inflammation or allergic and autoimmune pathogeneses. This suggests that the use of fusion proteins or Abs may be helpful in controlling these diseases, but their role and mechanism of action is not well defined. For example, GITR-Fc fusion protein binds GITRL with potentially 2 effects: 1) inhibition of GITR activation by endogenous GITRL, 2) activation of GITRL. In some models the first effect seemed prevalent, as when pleurisy was diminished by GITR-Fc injection[23]. In other models, the second effect was prevalent, as when mice injected with GITR-Fc developed an inflammatory reaction[28]. Moreover, in some experimental models such as *in vitro* stimulation of $GITR^{-/-}$ T lymphocyte or *C. albicans* infection, a higher activation in the absence of GITR has been described[26,95]. Although the mechanism of this effect was clarified only in part, the possibility that GITR/GITRL interaction regulates the activity of other cells in the system should be considered. Among these, a prominent role may be played by the regulation of APC function. In addition, in other models an antiapoptotic and protective function of GITR on peripheral tissue was described[31,60], suggesting that GITR triggering not always plays a pro-inflammatory role. Thus, the final response consequent to GITR/GITRL triggering may well depend on the experimental model used and most of the experimental designs warrant further studies, especially before planning the use of fusion proteins and Abs to modulate shock and the inflammatory process through the GITR/GITRL system.

### ACKNOWLEDGEMENTS

Associazione Italiana per la Ricerca sul Cancro (AIRC), Milan, supported this work.

## References

1. Nocentini, G., Giunchi, L., Ronchetti, S., Krausz, L.T., Bartoli, A., Moraca, R., Migliorati, G., and Riccardi, C. (1997) A new member of the tumor necrosis factor/nerve growth factor receptor family inhibits T cell receptor-induced apoptosis. *Proceedings of the National Academy of Sciences of the United States of America* **94**, 6216-6221.
2. Kim, J.D., Choi, B.K., Bae, J.S., Lee, U.H., Han, I.S., Lee, H.W., Youn, B.S., Vinay, D.S., and Kwon, B.S. (2003) Cloning and characterization of GITR ligand. *Genes Immun* **4**, 564-569.
3. Kwon, B., Yu, K.Y., Ni, J., Yu, G.L., Jang, I.K., Kim, Y.J., Xing, L., Liu, D., Wang, S.X., and Kwon, B.S. (1999) Identification of a novel activation-inducible protein of the tumor necrosis factor receptor superfamily and its ligand. *The Journal of biological chemistry* **274**, 6056-6061.
4. Gurney, A.L., Marsters, S.A., Huang, R.M., Pitti, R.M., Mark, D.T., Baldwin, D.T., Gray, A.M., Dowd, A.D., Brush, A.D., Heldens, A.D., Schow, A.D., Goddard, A.D., Wood, W.I., Baker, K.P., Godowski, P.J., and Ashkenazi, A. (1999) Identification of a new member of the tumor necrosis factor family and its receptor, a human ortholog of mouse GITR. *Curr Biol* **9**, 215-218.
5. Yu, K.Y., Kim, H.S., Song, S.Y., Min, S.S., Jeong, J.J., and Youn, B.S. (2003) Identification of a ligand for glucocorticoid-induced tumor necrosis factor receptor constitutively expressed in dendritic cells. *Biochem Biophys Res Commun* **310**, 433-438.
6. Ronchetti, S., Zollo, O., Bruscoli, S., Agostini, M., Bianchini, R., Nocentini, G., Ayroldi, E., and Riccardi, C. (2004) GITR, a member of the TNF receptor superfamily, is costimulatory to mouse T lymphocyte subpopulations. *Eur J Immunol* **34**, 613-622.
7. Tone, M., Tone, Y., Adams, E., Yates, S.F., Frewin, M.R., Cobbold, S.P., and Waldmann, H. (2003) Mouse glucocorticoid-induced tumor necrosis factor receptor ligand is costimulatory for T cells. *Proceedings of the National Academy of Sciences of the United States of America* **100**, 15059-15064.
8. Bossen, C., Ingold, K., Tardivel, A., Bodmer, J.L., Gaide, O., Hertig, S., Ambrose, C., Tschopp, J., and Schneider, P. (2006) Interactions of Tumor Necrosis Factor (TNF) and TNF Receptor Family Members in the Mouse and Human. *The Journal of biological chemistry* **281**, 13964-13971.
9. Nocentini, G., and Riccardi, C. (2005) GITR: a multifaceted regulator of immunity belonging to the tumor necrosis factor receptor superfamily. *Eur J Immunol* **35**, 1016-1022.
10. Esparza, E.M., and Arch, R.H. (2006) Signaling triggered by glucocorticoid-induced tumor necrosis factor receptor family-related gene: regulation at the interface between regulatory T cells and immune effector cells. *Front Biosci* **11**, 1448-1465.
11. Shevach, E.M., and Stephens, G.L. (2006) The GITR-GITRL interaction: co-stimulation or contrasuppression of regulatory activity? *Nat Rev Immunol* **6**, 613-618.
12. Hanabuchi, S., Watanabe, N., Wang, Y.H., Wang, Y.H., Ito, T., Shaw, J., Cao, W., Qin, F.X., and Liu, Y.J. (2006) Human plasmacytoid predendritic cells activate NK cells through glucocorticoid-induced tumor necrosis factor receptor-ligand (GITRL). *Blood* **107**, 3617-3623.
13. McHugh, R.S., Whitters, M.J., Piccirillo, C.A., Young, D.A., Shevach, E.M., Collins, M., and Byrne, M.C. (2002) CD4(+)CD25(+) immunoregulatory T cells: gene expression analysis reveals a functional role for the glucocorticoid-induced TNF receptor. *Immunity* **16**, 311-323.
14. Kohm, A.P., Williams, J.S., and Miller, S.D. (2004) Cutting edge: ligation of the glucocorticoid-induced TNF receptor enhances autoreactive CD4+ T cell activation and experimental autoimmune encephalomyelitis. *J Immunol* **172**, 4686-4690.
15. Kanamaru, F., Youngnak, P., Hashiguchi, M., Nishioka, T., Takahashi, T., Sakaguchi, S., Ishikawa, I., and Azuma, M. (2004) Costimulation via glucocorticoid-induced TNF receptor in both conventional and CD25+ regulatory CD4+ T cells. *J Immunol* **172**, 7306-7314.
16. Stephens, G.L., McHugh, R.S., Whitters, M.J., Young, D.A., Luxenberg, D., Carreno, B.M., Collins, M., and Shevach, E.M. (2004) Engagement of glucocorticoid-induced TNFR family-related receptor on effector T cells by its ligand mediates resistance to suppression by CD4+CD25+ T cells. *J Immunol* **173**, 5008-5020.
17. Zelenika, D., Adams, E., Humm, S., Graca, L., Thompson, S., Cobbold, S.P., and Waldmann, H. (2002) Regulatory T cells overexpress a subset of Th2 gene transcripts. J. Immunol. **168**, 1069-1079.
18. Uraushihara, K., Kanai, T., Ko, K., Totsuka, T., Makita, S., Iiyama, R., Nakamura, T., and Watanabe, M. (2003) Regulation of murine inflammatory bowel disease by CD25+ and CD25- CD4+ glucocorticoid-induced TNF receptor family-related gene+ regulatory T cells. **171**, 708-716.
19. Shimizu, J., Yamazaki, S., Takahashi, T., Ishida, Y., and Sakaguchi, S. (2002) Stimulation of CD25(+)CD4(+) regulatory T cells through GITR breaks immunological self-tolerance. *Nat Immunol* **3**, 135-142.

20. Scotto, L., Naiyer, A.J., Galluzzo, S., Rossi, P., Manavalan, J.S., Kim-Schulze, S., Fang, J., Favera, R.D., Cortesini, R., and Suciu-Foca, N. (2004) Overlap between molecular markers expressed by naturally occurring CD4+CD25+ regulatory T cells and antigen specific CD4+CD25+ and CD8+CD28- T suppressor cells. *Hum Immunol* **65**, 1297-1306.
21. Cosmi, L., Liotta, F., Lazzeri, E., Francalanci, M., Angeli, R., Mazzinghi, B., Santarlasci, V., Manetti, R., Vanini, V., Romagnani, P., Maggi, E., Romagnani, S., and Annunziato, F. (2003) Human CD8+CD25+ thymocytes share phenotypic and functional features with CD4+CD25+ regulatory thymocytes. *Blood* **102**, 4107-4114.
22. Kim, H.J., Kim, H.Y., Kim, B.K., Kim, S., and Chung, D.H. (2006) Engagement of glucocorticoid-induced TNF receptor costimulates NKT cell activation *in vitro* and *in vivo*. *J Immunol* **176**, 3507-3515.
23. Cuzzocrea, S., Nocentini, G., Di Paola, R., Agostini, M., Mazzon, E., Ronchetti, S., Crisafulli, C., Esposito, E., Caputi, A.P., and Riccardi, C. (2006) Proinflammatory role of glucocorticoid-induced TNF receptor-related gene in acute lung inflammation. *J Immunol* **177**, 631-641.
24. Santucci, L., Agostini, M., Bruscoli, S., Mencarelli, A., Ronchetti, S., Ayroldi, E., Morelli, A., Baldoni, M., and Riccardi, C. (2007) GITR modulates innate and adaptive mucosal immunity during the development of experimental colitis in mice. *Gut* **56**, 52-60.
25. Nakae, S., Suto, H., Iikura, M., Kakurai, M., Sedgwick, J.D., Tsai, M., and Galli, S.J. (2006) Mast cells enhance T cell activation: importance of mast cell costimulatory molecules and secreted TNF. *J Immunol* **176**, 2238-2248.
26. Ronchetti, S., Nocentini, G., Riccardi, C., and Pandolfi, P.P. (2002) Role of GITR in activation response of T lymphocytes. *Blood* **100**, 350-352.
27. Ji, H.B., Liao, G., Faubion, W.A., Abadia-Molina, A.C., Cozzo, C., Laroux, F.S., Caton, A., and Terhorst, C. (2004) Cutting edge: the natural ligand for glucocorticoid-induced TNF receptor-related protein abrogates regulatory T cell suppression. *J Immunol* **172**, 5823-5827.
28. Shin, H.H., Kim, S.G., Lee, M.H., Suh, J.H., Kwon, B.S., and Choi, H.S. (2003) Soluble glucocorticoid-induced TNF receptor (sGITR) induces inflammation in mice. *Exp Mol Med* **35**, 358-364.
29. Shin, H.H., Kwon, B.S., and Choi, H.S. (2002) Recombinant glucocorticoid induced tumour necrosis factor receptor (rGITR) induced COX-2 activity in murine macrophage Raw 264.7 cells. *Cytokine* **19**, 187-192.
30. Albers, A.E., Ferris, R.L., Kim, G.G., Chikamatsu, K., DeLeo, A.B., and Whiteside, T.L. (2005) Immune responses to p53 in patients with cancer: enrichment in tetramer+ p53 peptide-specific T cells and regulatory T cells at tumor sites. *Cancer Immunol Immunother* **54**, 1072-1081.
31. Wang, J., Devgan, V., Corrado, M., Prabhu, N.S., El-Deiry, W.S., Riccardi, C., Pandolfi, P.P., Missero, C., and Dotto, G.P. (2005) Glucocorticoid-induced tumor necrosis factor receptor is a p21Cip1/WAF1 transcriptional target conferring resistance of keratinocytes to UV light-induced apoptosis. *The Journal of Biological Chemistry* **280**, 37725-37731.
32. Shin, H.H., Kim, S.J., Lee, D.S., and Choi, H.S. (2005) Soluble glucocorticoid-induced tumor necrosis factor receptor (sGITR) stimulates osteoclast differentiation in response to receptor activator of NF-kappaB ligand (RANKL) in osteoclast cells. *Bone* **36**, 832-839.
33. Kim, B.J., Li, Z., Fariss, R.N., Shen de, F., Mahesh, S.P., Egwuagu, C., Yu, C.R., Nagineni, C.N., Chan, C.C., and Nussenblatt, R.B. (2004) Constitutive and cytokine-induced GITR ligand expression on human retinal pigment epithelium and photoreceptors. *Invest Ophthalmol Vis Sci* **45**, 3170-3176.
34. Boussaud, V., Soler, P., Moreau, J., Goodwin, R.G., and Hance, A.J. (1998) Expression of three members of the TNF-R family of receptors (4-1BB, lymphotoxin-beta receptor, and Fas) in human lung. *Eur Respir J* **12**, 926-931.
35. Li, Z., Mahesh, S.P., Kim, B.J., Buggage, R.R., and Nussenblatt, R.B. (2003) Expression of glucocorticoid induced TNF receptor family related protein (GITR) on peripheral T cells from normal human donors and patients with non-infectious uveitis. *J Autoimmun* **21**, 83-92.
36. Zhang, X., Chen, Z., Huang, H., Gordon, J.R., and Xiang, J. (2002) DNA microarray analysis of the gene expression profiles of naive versus activated tumor-specific T cells. *Life Sci* **71**, 3005-3017.
37. Siepmann, K., Biester, S., Plskova, J., Muckersie, E., Duncan, L., and Forrester, J.V. (2006) CD4(+)CD25 (+) T regulatory cells induced by LPS-activated bone marrow dendritic cells suppress experimental autoimmune uveoretinitis *in vivo*. *Graefes Arch. Clin. Exp. Ophthalmol.* **245**, 221-229.
38. Sakaguchi, S., Sakaguchi, N., Asano, M., Itoh, M., and Toda, M. (1995) Immunologic self-tolerance maintained by activated T cells expressing IL-2 receptor alpha-chains (CD25). Breakdown of a single mechanism of self-tolerance causes various autoimmune diseases. *J Immunol* **155**, 1151-1164.
39. Herman, A.E., Freeman, G.J., Mathis, D., and Benoist, C. (2004) CD4+CD25+ T regulatory cells dependent on ICOS promote regulation of effector cells in the prediabetic lesion. *The Journal of Experimental Medicine* **199**, 1479-1489.
40. Levings, M.K., Sangregorio, R., Sartirana, C., Moschin, A.L., Battaglia, M., Orban, P.C., and Roncarolo, M.G. (2002) Human CD25+CD4+ T suppressor cell clones produce transforming growth factor beta, but not interleukin 10, and are distinct from type 1 T regulatory cells. *The Journal of Experimental Medicine* **196**, 1335-1346.
41. Cobbold, S.P., Castejon, R., Adams, E., Zelenika, D., Graca, L., Humm, S., and Waldmann, H. (2004) Induction of foxP3+ regulatory T cells in the periphery of T cell receptor transgenic mice tolerized to transplants. J. Immunol. **172**, 6003-6010.
42. Heikkinen, J., Mottonen, M., Alanen, A., and Lassila, O. (2004) Phenotypic characterization of regulatory T cells in the human decidua. Clin. Exp. Immunol. **136**, 373-378.

43. de Kleer, I.M., Wedderburn, L.R., Taams, L.S., Patel, A., Varsani, H., Klein, M., de Jager, W., Pugayung, G., Giannoni, F., Rijkers, G., Albani, S., Kuis, W., and Prakken, B. (2004) CD4+CD25bright regulatory T cells .actively regulate inflammation in the joints of patients with the remitting form of juvenile idiopathic arthritis. J. Immunol. **172**, 6435-6443.
44. Makita, S., Kanai, T., Oshima, S., Uraushihara, K., Totsuka, T., Sawada, T., Nakamura, T., Koganei, K., Fukushima, T., and Watanabe, M. (2004) CD4+CD25bright T cells in human intestinal lamina propria as regulatory cells. *J Immunol* **173**, 3119-3130.
45. Fontenot, J.D., Gavin, M.A., and Rudensky, A.Y. (2003) Foxp3 programs the development and function of CD4+CD25+ regulatory T cells. *Nat Immunol* **4**, 330-336.
46. Chen, C., Rowell, E.A., Thomas, R.M., Hancock, W.W., and Wells, A.D. (2006) Transcriptional regulation by foxp3 is associated with direct promoter occupancy and modulation of histone acetylation. *The Journal of Biological Chemistry* **281**, 36828-36834.
47. Khattri, R., Cox, T., Yasayko, S.A., and Ramsdell, F. (2003) An essential role for Scurfin in CD4+CD25+ T regulatory cells. *Nat Immunol* **4**, 337-342.
48. Hori, S., Nomura, T., and Sakaguchi, S. (2003) Control of regulatory T cell development by the transcription factor Foxp3. *Science* **299**, 1057-1061.
49. Veldman, C., Pahl, A., Beissert, S., Hansen, W., Buer, J., Dieckmann, D., Schuler, G., and Hertl, M. (2006) Inhibition of the transcription factor Foxp3 converts desmoglein 3-specific type 1 regulatory T cells into Th2-like cells. *J Immunol* **176**, 3215-3222.
50. Ono, M., Shimizu, J., Miyachi, Y., and Sakaguchi, S. (2006) Control of autoimmune myocarditis and multiorgan inflammation by glucocorticoid-induced TNF receptor family-related protein(high), Foxp3-expressing CD25+ and CD25- regulatory T cells. *J Immunol* **176**, 4748-4756.
51. Krupnick, A.S., Gelman, A.E., Barchet, W., Richardson, S., Kreisel, F.H., Turka, L.A., Colonna, M., Patterson, G.A., and Kreisel, D. (2005) Murine vascular endothelium activates and induces the generation of allogeneic CD4+25+Foxp3+ regulatory T cells. *J Immunol* **175**, 6265-6270.
52. Grimbert, P., Bouguermouh, S., Baba, N., Nakajima, T., Allakhverdi, Z., Braun, D., Saito, H., Rubio, M., Delespesse, G., and Sarfati, M. (2006) Thrombospondin/CD47 interaction: a pathway to generate regulatory T cells from human CD4+ CD25- T cells in response to inflammation. *J Immunol* **177**, 3534-3541.
53. Gavin, M., and Rudensky, A. (2003) Control of immune homeostasis by naturally arising regulatory CD4+ T cells. *Curr Opin Immunol* **15**, 690-696.
54. Every, A.L., Kramer, D.R., Mannering, S.I., Lew, A.M., and Harrison, L.C. (2006) Intranasal vaccination with proinsulin DNA induces regulatory CD4+ T cells that prevent experimental autoimmune diabetes. *J Immunol* **176**, 4608-4615.
55. Chen, X., Murakami, T., Oppenheim, J.J., and Howard, O.M. (2004) Differential response of murine CD4+CD25+ and CD4+CD25- T cells to dexamethasone-induced cell death. *Eur J Immunol* **34**, 859-869.
56. Zhan, Y., Funda, D.P., Every, A.L., Fundova, P., Purton, J.F., Liddicoat, D.R., Cole, T.J., Godfrey, D.I., Brady, J.L., Mannering, S.I., Harrison, L.C., and Lew, A.M. (2004) TCR-mediated activation promotes GITR upregulation in T cells and resistance to glucocorticoid-induced death. *Int Immunol* **16**, 1315-1321.
57. Nardelli, B., Zaritskaya, L., McAuliffe, W., Ni, Y., Lincoln, C., Cho, Y.H., Birse, C.E., Halpern, W., Ullrich, S., and Moore, P.A. (2006) Osteostat/tumor necrosis factor superfamily 18 inhibits osteoclastogenesis and is selectively expressed by vascular endothelial cells. *Endocrinology* **147**, 70-78.
58. Mahesh, S.P., Li, Z., Liu, B., Fariss, R.N., and Nussenblatt, R.B. (2006) Expression of GITR ligand abrogates immunosuppressive function of ocular tissue and differentially modulates inflammatory cytokines and chemokines. *Eur J Immunol* **36**, 2128-2138.
59. Cardona, I.D., Goleva, E., Ou, L.S., and Leung, D.Y. (2006) Staphylococcal enterotoxin B inhibits regulatory T cells by inducing glucocorticoid-induced TNF receptor-related protein ligand on monocytes. *J Allergy Clin Immunol* **117**, 688-695.
60. Suvas, S., Kim, B., Sarangi, P.P., Tone, M., Waldmann, H., and Rouse, B.T. (2005) In vivo kinetics of GITR and GITR ligand expression and their functional significance in regulating viral immunopathology. *J Virol* **79**, 11935-11942.
61. Cuzzocrea, S., Nocentini, G., Di Paola, R., Mazzon, E., Ronchetti, S., Genovese, T., Muia, C., Caputi, A.P., and Riccardi, C. (2004) Glucocorticoid-induced TNF receptor family gene (GITR) knockout mice exhibit a resistance to splanchnic artery occlusion (SAO) shock. *Journal of Leukocyte Biology* **76**, 933-940.
62. Lee, H.S., Shin, H.H., Kwon, B.S., and Choi, H.S. (2003) Soluble glucocorticoid-induced tumor necrosis factor receptor (sGITR) increased MMP-9 activity in murine macrophage. *J Cell Biochem* **88**, 1048-1056.
63. Cuzzocrea, S., Ronchetti, S., Genovese, T., Mazzon, E., Agostini, M., Di Paola, R., Esposito, E., Muia, C., Nocentini, G., and Riccardi, C. (2007) Genetic and pharmacological inhibition of GITR-GITRL interaction reduces chronic lung injury induced by bleomycin instillation. *Faseb J.* **21**, 117-129.
64. Clark, R.A., and Kupper, T.S. (2007) IL-15 and dermal fibroblasts induce proliferation of natural regulatory T cells isolated from human skin. *Blood* **109**, 194-202.
65. Cuzzocrea, S., Ayroldi, E., Di Paola, R., Agostini, M., Mazzon, E., Bruscoli, S., Genovese, T., Ronchetti, S.,

Caputi, A.P., and Riccardi, C. (2005) Role of glucocorticoid-induced TNF receptor family gene (GITR) in collagen-induced arthritis. *Faseb J* **19**, 1253-1265.
66. Patel, M., Xu, D., Kewin, P., Choo-Kang, B., McSharry, C., Thomson, N.C., and Liew, F.Y. (2005) Glucocorticoid-induced TNFR family-related protein (GITR) activation exacerbates murine asthma and collagen-induced arthritis. *Eur J Immunol* **35**, 3581-3590.
67. Mottonen, M., Heikkinen, J., Mustonen, L., Isomaki, P., Luukkainen, R., and Lassila, O. (2005) CD4+ CD25+ T cells with the phenotypic and functional characteristics of regulatory T cells are enriched in the synovial fluid of patients with rheumatoid arthritis. *Clin Exp Immunol* **140**, 360-367.
68. Morris, G.P., and Kong, Y.C. (2006) Interference with CD4+CD25+ T-cell-mediated tolerance to experimental autoimmune thyroiditis by glucocorticoid-induced tumor necrosis factor receptor monoclonal antibody. *J Autoimmun* **26**, 24-31.
69. Gruss, H.J., and Dower, S.K. (1995) The TNF ligand superfamily and its relevance for human diseases. *Cytokines and Molecular Therapy* **1**, 75-105.
70. Dittmer, U., He, H., Messer, R.J., Schimmer, S., Olbrich, A.R., Ohlen, C., Greenberg, P.D., Stromnes, I.M., Iwashiro, M., Sakaguchi, S., Evans, L.H., Peterson, K.E., Yang, G., and Hasenkrug, K.J. (2004) Functional impairment of CD8(+) T cells by regulatory T cells during persistent retroviral infection. *Immunity* **20**, 293-303.
71. La, S., Kim, E., and Kwon, B. (2005) In vivo ligation of glucocorticoid-induced TNF receptor enhances the T-cell immunity to herpes simplex virus type 1. *Exp Mol Med* **37**, 193-198.
72. Smith, C.A., Farrah, T., and Goodwin, R.G. (1994) The TNF receptor superfamily of cellular and viral proteins: activation, costimulation, and death. *Cell* **76**, 959-962.
73. Shin, H.H., Kim, S.J., Lee, H.S., and Choi, H.S. (2004) The soluble glucocorticoid-induced tumor necrosis factor receptor causes cell cycle arrest and apoptosis in murine macrophages. *Biochem Biophys Res Commun* **316**, 24-32.
74. Yang, L., Froio, R.M., Sciuto, T.E., Dvorak, A.M., Alon, R., and Luscinskas, F.W. (2005) ICAM-1 regulates neutrophil adhesion and transcellular migration of TNF-alpha-activated vascular endothelium under flow. *Blood* **106**, 584-592.
75. Cuzzocrea, S., and Reiter, R.J. (2001) Pharmacological action of melatonin in shock, inflammation and ischemia/reperfusion injury. *European Journal of Pharmacology* **426**, 1-10.
76. Vane, J.R., and Botting, R.M. (1995) New insights into the mode of action of anti-inflammatory drugs. *Inflamm Res* **44**, 1-10.
77. Shin, H.H., Kim, S.J., Kang, S.Y., Lee, D.S., and Choi, H.S. (2006) Soluble glucocorticoid-induced tumor necrosis factor receptor stimulates osteoclastogenesis by down-regulation of osteoprotegerin in bone marrow stromal cells. *Bone* **39**, 716-723.
78. Shin, H.H., Lee, H.W., and Choi, H.S. (2003) Induction of nitric oxide synthase (NOS) by soluble glucocorticoid induced tumor necrosis factor receptor (sGITR) is modulated by IFN-gamma in murine macrophage. *Exp Mol Med* **35**, 175-180.
79. Shin, H.H., Lee, M.H., Kim, S.G., Lee, Y.H., Kwon, B.S., and Choi, H.S. (2002) Recombinant glucocorticoid induced tumor necrosis factor receptor (rGITR) induces NOS in murine macrophage. *FEBS Lett* **514**, 275-280.
80. Kim, W.J., Bae, E.M., Kang, Y.J., Bae, H.U., Hong, S.H., Lee, J.Y., Park, J.E., Kwon, B.S., Suk, K., and Lee, W.H. (2006) Glucocorticoid-induced tumour necrosis factor receptor family related protein (GITR) mediates inflammatory activation of macrophages that can destabilize atherosclerotic plaques. *Immunology* **119**, 421-429.
81. Kim, S.J., Shin, H.H., Park, S.Y., Lee, D.S., Lee, E.A., Cho, S.D., Cho, H.R., Miyazawa, K., and Choi, H.S. (2006) Induction of MMP-13 expression by soluble human glucocorticoid-induced tumor necrosis factor receptor in fibroblast-like synovial cells. Osteoarthritis and cartilage / OARS, *Osteoarthritis Research Society* **14**, 146-153.
82. Schaub, B., Campo, M., He, H., Perkins, D., Gillman, M.W., Gold, D.R., Weiss, S., Lieberman, E., and Finn, P.W. (2006) Neonatal immune responses to TLR2 stimulation: influence of maternal atopy on Foxp3 and IL-10 expression. *Respir. Res.* **7**, 40.
83. Esparza, E.M., and Arch, R.H. (2005) Glucocorticoid-induced TNF receptor functions as a costimulatory receptor that promotes survival in early phases of T cell activation. *J Immunol* **174**, 7869-7874.
84. Kohm, A.P., Podojil, J.R., Williams, J.S., McMahon, J.S., and Miller, S.D. (2005) CD28 regulates glucocorticoid-induced TNF receptor family-related gene expression on CD4+ T cells via IL-2-dependent mechanisms. *Cell Immunol* **235**, 56-64.
85. Kim, J., Choi, W.S., Kang, H., Kim, H.J., Suh, J.H., Sakaguchi, S., and Kwon, B. (2006) Conversion of alloantigen-specific CD8+ T cell anergy to CD8+ T cell priming through in vivo ligation of glucocorticoid-induced TNF receptor. *J Immunol* **176**, 5223-5231.
86. Muriglan, S.J., Ramirez-Montagut, T., Alpdogan, O., Van Huystee, T.W., Eng, J.M., Hubbard, V.M., Kochman, A.A., Tjoe, K.H., Riccardi, C., Pandolfi, P.P., Sakaguchi, S., Houghton, A.N., and Van Den Brink, M.R. (2004) GITR activation induces an opposite effect on alloreactive CD4(+) and CD8(+) T cells in graft-versus-host disease. *The Journal of Experimental Medicine* **200**, 149-157.
87. Shimizu, J., and Moriizumi, E. (2003) CD4+CD25- T cells in aged mice are hyporesponsive and exhibit suppressive activity. *J Immunol* **170**, 1675-1682.
88. Tsaknaridis, L., Spencer, L., Culbertson, N., Hicks, K., LaTocha, D., Chou, Y.K., Whitham, R.H., Bakke, A., Jones, R.E., Offner, H., Bourdette, D.N., and Vandenbark, A.A. (2003) Functional assay for human CD4+CD25+

Treg cells reveals an age-dependent loss of suppressive activity. *J Neurosci Res* **74**, 296-308.
89. La Cava, A., Ebling, F.M., and Hahn, B.H. (2004) Ig-reactive CD4+CD25+ T cells from tolerized (New Zealand Black x New Zealand White)F1 mice suppress in vitro production of antibodies to DNA. *J Immunol* **173**, 3542-3548.
90. Valzasina, B., Guiducci, C., Dislich, H., Killeen, N., Weinberg, A.D., and Colombo, M.P. (2005) Triggering of OX40 (CD134) on CD4(+)CD25+ T cells blocks their inhibitory activity: a novel regulatory role for OX40 and its comparison with GITR. *Blood* **105**, 2845-2851.
91. Gondek, D.C., Lu, L.F., Quezada, S.A., Sakaguchi, S., and Noelle, R.J. (2005) Cutting edge: contact-mediated suppression by CD4+CD25+ regulatory cells involves a granzyme B-dependent, perforin-independent mechanism. *J Immunol* **174**, 1783-1786.
92. Ramirez-Montagut, T., Chow, A., Hirschhorn-Cymerman, D., Terwey, T.H., Kochman, A.A., Lu, S., Miles, R.C., Sakaguchi, S., Houghton, A.N., and van den Brink, M.R. (2006) Glucocorticoid-Induced TNF Receptor Family Related Gene Activation Overcomes Tolerance/Ignorance to Melanoma Differentiation Antigens and Enhances Antitumor Immunity. *J Immunol* **176**, 6434-6442.
93. Ko, K., Yamazaki, S., Nakamura, K., Nishioka, T., Hirota, K., Yamaguchi, T., Shimizu, J., Nomura, T., Chiba, T., and Sakaguchi, S. (2005) Treatment of advanced tumors with agonistic anti-GITR mAb and its effects on tumor-infiltrating Foxp3+CD25+CD4+ regulatory T cells. *The Journal of Experimental Medicine* **202**, 885-891.
94. He, H., Messer, R.J., Sakaguchi, S., Yang, G., Robertson, S.J., and Hasenkrug, K.J. (2004) Reduction of retrovirus-induced immunosuppression by *in vivo* modulation of T cells during acute infection. *J Virol* **78**, 11641-11647.
95. Agostini, M., Cenci, E., Pericolini, E., Nocentini, G., Bistoni, G., Vecchiarelli, A., and Riccardi, C. (2005) The glucocorticoid-induced tumor necrosis factor receptor-related gene modulates the response to Candida albicans infection. *Infect Immun* **73**, 7502-7508.
96. Nocentini, G., Bartoli, A., Ronchetti, S., Giunchi, L., Cupelli, A., Delfino, D., Migliorati, G., and Riccardi, C. (2000) Gene structure and chromosomal assignment of mouse GITR, a member of the tumor necrosis factor/nerve growth factor receptor family. *DNA Cell Biol* **19**, 205-217.
97. Banner, D.W., D'Arcy, A., Janes, W., Gentz, R., Schoenfeld, H.J., Broger, C., Loetscher, H., and Lesslauer, W. (1993) Crystal structure of the soluble human 55 kd TNF receptor-human TNF beta complex: implications for TNF receptor activation. *Cell* **73**, 431-445.
98. Jones, M.D., Hunt, J., Liu, J.L., Patterson, S.D., Kohno, T., and Lu, H.S. (1997) Determination of tumor necrosis factor binding protein disulfide structure: deviation of the fourth domain structure from the TNFR/NGFR family cysteine-rich region signature. *Biochemistry* **36**, 14914-14923.
99. Orlinick, J.R., Vaishnaw, A., Elkon, K.B., and Chao, M.V. (1997) Requirement of cysteine-rich repeats of the Fas receptor for binding by the Fas ligand. *The Journal of Biological Chemistry* **272**, 28889-28894.
100. Welcher, A.A., Bitler, C.M., Radeke, M.J., and Shooter, E.M. (1991) Nerve growth factor binding domain of the nerve growth factor receptor. *Proceedings of the National Academy of Sciences of the United States of America* **88**, 159-163.
101. Spinicelli, S., Nocentini, G., Ronchetti, S., Krausz, L.T., Bianchini, R., and Riccardi, C. (2002) GITR interacts with the pro-apoptotic protein Siva and induces apoptosis. *Cell Death Differ* **9**, 1382-1384.
102. Lotz, M., Setareh, M., von Kempis, J., and Schwarz, H. (1996) The nerve growth factor/tumor necrosis factor receptor family. *Journal of Leukocyte Biology* **60**, 1-7.
103. Screaton, G.R., Xu, X.N., Olsen, A.L., Cowper, A.E., Tan, R., McMichael, A.J., and Bell, J.I. (1997) LARD: a new lymphoid-specific death domain containing receptor regulated by alternative pre-mRNA splicing. *Proceedings of the National Academy of Sciences of the United States of America* **94**, 4615-4619.
104. Nocentini, G., Ronchetti, S., Bartoli, A., Spinicelli, S., Delfino, D., Brunetti, L., Migliorati, G., and Riccardi, C. (2000) Identification of three novel mRNA splice variants of GITR. ***Cell Death Differ.*** **7**, 408-410.
105. Hauer, J., Puschner, S., Ramakrishnan, P., Simon, U., Bongers, M., Federle, C., and Engelmann, H. (2005) TNF receptor (TNFR)-associated factor (TRAF) 3 serves as an inhibitor of TRAF2/5-mediated activation of the noncanonical NF-kappaB pathway by TRAF-binding TNFRs. *Proceedings of the National Academy of Sciences of the United States of America* **102**, 2874-2879.
106. Park, Y.C., Burkitt, V., Villa, A.R., Tong, L., and Wu, H. (1999) Structural basis for self-association and receptor recognition of human TRAF2. *Nature* **398**, 533-538.
107. Ye, H., Arron, J.R., Lamothe, B., Cirilli, M., Kobayashi, T., Shevde, N.K., Segal, D., Dzivenu, O.K., Vologodskaia, M., Yim, M., Du, K., Singh, S., Pike, J.W., Darnay, B.G., Choi, Y., and Wu, H. (2002) Distinct molecular mechanism for initiating TRAF6 signalling. *Nature* **418**, 443-447.
108. Esparza, E.M., and Arch, R.H. (2005) Glucocorticoid-induced TNF receptor, a costimulatory receptor on naive and activated T cells, uses TNF receptor-associated factor 2 in a novel fashion as an inhibitor of NF-kappa B activation. ***J. Immunol.*** **174**, 7875-7882.
109. Esparza, E.M., Lindsten, T., Stockhausen, J.M., and Arch, R.H. (2006) Tumor necrosis factor receptor (TNFR)-associated factor 5 is a critical intermediate of costimulatory signaling pathways triggered by glucocorticoid-induced TNFR in T cells. *The Journal of Biological Chemistry* **281**, 8559-8564.
110. Prasad, K.V., Ao, Z., Yoon, Y., Wu, M.X., Rizk, M., Jacquot, S., and Schlossman, S.F. (1997) CD27, a member of the tumor necrosis factor receptor family, induces apoptosis and binds to Siva, a proapoptotic protein. *Proceedings*

*of the National Academy of Sciences of the United States of America* **94**, 6346-6351.
111. Py, B., Slomianny, C., Auberger, P., Petit, P.X., and Benichou, S. (2004) Siva-1 and an alternative splice form lacking the death domain, Siva-2, similarly induce apoptosis in T lymphocytes via a caspase-dependent mitochondrial pathway. *J Immunol* **172**, 4008-4017.
112. Lu, L.F., Gondek, D.C., Scott, Z.A., and Noelle, R.J. (2005) NF kappa B-inducing kinase deficiency results in the development of a subset of regulatory T cells, which shows a hyperproliferative activity upon glucocorticoid-induced TNF receptor family-related gene stimulation. *J Immunol* **175**, 1651-1657.
113. Ramakrishnan, P., Wang, W., and Wallach, D. (2004) Receptor-specific signaling for both the alternative and the canonical NF-kappaB activation pathways by NF-kappaB-inducing kinase. *Immunity* **21**, 477-489.
114. Dejardin, E. (2006) The alternative NF-kappaB pathway from biochemistry to biology: pitfalls and promises for future drug development. *Biochemical pharmacology* **72**, 1161-1179.
115. Gudi, R., Barkinge, J., Hawkins, S., Chu, F., Manicassamy, S., Sun, Z., Duke-Cohan, J.S., and Prasad, K.V. (2006) Siva-1 negatively regulates NF-kappaB activity: effect on T-cell receptor-mediated activation-induced cell death (AICD). *Oncogene* **25**, 3458-3462.
116. Tirone, F. (2001) The gene PC3 (TIS21/BTG2), prototype member of the PC3/BTG/TOB family: regulator in control of cell growth, differentiation, and DNA repair? *Journal of Cellular Physiology* **187**, 155-165.
117. Lin, W.J., Gary, J.D., Yang, M.C., Clarke, S., and Herschman, H.R. (1996) The mammalian immediate-early TIS21 protein and the leukemia-associated BTG1 protein interact with a protein-arginine N-methyltransferase. *The Journal of Biological Chemistry* **271**, 15034-15044.
118. An, W., Kim, J., and Roeder, R.G. (2004) Ordered cooperative functions of PRMT1, p300, and CARM1 in transcriptional activation by p53. *Cell* **117**, 735-748.
119. Berthet, C., Guehenneux, F., Revol, V., Samarut, C., Lukaszewicz, A., Dehay, C., Dumontet, C., Magaud, J.P., and Rouault, J.P. (2002) Interaction of PRMT1 with BTG/TOB proteins in cell signalling: molecular analysis and functional aspects. *Genes Cells* **7**, 29-39.
120. Herrmann, F., Lee, J., Bedford, M.T., and Fackelmayer, F.O. (2005) Dynamics of human protein arginine methyltransferase 1(PRMT1) *in vivo*. *The Journal of Biological Chemistry* **280**, 38005-38010.
121. Mowen, K.A., Schurter, B.T., Fathman, J.W., David, M., and Glimcher, L.H. (2004) Arginine methylation of NIP45 modulates cytokine gene expression in effector T lymphocytes. *Molecular cell* **15**, 559-571.
122. Ganesh, L., Yoshimoto, T., Moorthy, N.C., Akahata, W., Boehm, M., Nabel, E.G., and Nabel, G.J. (2006) Protein methyltransferase 2 inhibits NF-kappa B function and promotes apoptosis. *Molecular and Cellular Biology* **26**, 3864-3874.
123. Osanai, T., Saitoh, M., Sasaki, S., Tomita, H., Matsunaga, T., and Okumura, K. (2003) Effect of shear stress on asymmetric dimethylarginine release from vascular endothelial cells. *Hypertension* **42**, 985-990.
124. Lieberson, R., Mowen, K.A., McBride, K.D., Leautaud, V., Zhang, X., Suh, W.K., Wu, L., and Glimcher, L.H. (2001) Tumor necrosis factor receptor-associated factor (TRAF) 2 represses the T helper cell type 2 response through interaction with NFAT-interacting protein (NIP45). *The Journal of Experimental Medicine* **194**, 89-98.
125. So, T., Salek-Ardakani, S., Nakano, H., Ware, C.F., and Croft, M. (2004) TNF receptor-associated factor 5 limits the induction of Th2 immune responses. *J Immunol* **172**, 4292-4297.
